# Modeling the spatial resolution of magnetic solitons in magnetic force microscopy and the effect on their sizes

**DOI:** 10.1038/s41598-025-95584-9

**Published:** 2025-04-08

**Authors:** I. Castro, A. Riveros, J. L. Palma, L. Abelmann, R. Tomasello, D. R. Rodrigues, A. Giordano, G. Finocchio, R. A. Gallardo, N. Vidal-Silva

**Affiliations:** 1 Departamento de Ciencias Físicas, Universidad de La Frontera, Casilla 54-D, 4811186 Temuco, Chile; 2 Centro de Investigación en Ingeniería de Materiales, FINARQ, Universidad Central de Chile, Avda. Santa Isabel 1186, 8330601 Santiago, Chile; 3 Center for the Development of Nanoscience and Nanotechnology (CEDENNA), 9170124 Santiago, Chile; 4 Delft University of Technology, Delft, The Netherlands; 5 Department of Electrical and Information Engineering, Technical University of Bari, 70125 Bari, Italy; 6 Department of Engineering, University of Messina, 98166 Messina, Italy; 7 Department of Mathematical and Computer Sciences, Physical Sciences and Earth Sciences, University of Messina, 98166 Messina, Italy; 8 Universidad Técnica Federico Santa María, Avenida España 1680, 2390123 Valparaiso, Chile

**Keywords:** Condensed-matter physics, Techniques and instrumentation

## Abstract

In this work, we explored theoretically the spatial resolution of magnetic solitons and the variations of their sizes when subjected to a magnetic force microscopy (MFM) measurement. Next to tip-sample separation, we considered reversal in the magnetization direction of the tip, showing that the magnetic soliton size measurement can be strongly affected by the magnetization direction of the tip. In addition to previous studies that only consider thermal fluctuations, we developed a theoretical method to obtain the minimum observable length of a magnetic soliton and its length variation due to the influence of the MFM tip by minimizing the soliton’s magnetic energy. We show that a simple spherical model for the MFM tip can capture most of the physics underlying tip-sample interactions, with the key requirement being an estimate of the magnetization field within the sample. Our model uses analytical and numerical calculations and prevents overestimating the characteristic length scales from MFM images. We compared our method with available data from MFM measurements of domain wall widths, and we performed micromagnetic simulations of a skyrmion-tip system, finding a good agreement for both attractive and repulsive domain wall profile signals and for the skyrmion diameter in the presence of the magnetic tip. In addition, the theoretically calculated frequency shift presents good qualitative agreement with experimental measurements. Our results provide significant insights for a better interpretation of MFM measurements of different magnetic solitons and will be helpful in the design of potential reading devices based on magnetic solitons as information carriers.

## Introduction

The goal of miniaturizing magnetic elements in efficient micro- and nanodevices is driving advances in synthesis and characterization techniques. Magnetic force microscopy (MFM)^1–5^ stands out as a versatile and straightforward method for imaging magnetic textures within samples, as evidenced by various studies^6–11^. Its working principle is based on the magnetic interaction between a small magnetic element (the tip) mounted on a cantilever spring, and a magnetic sample, which should be thin enough to consider no significant variations of the sample’s magnetization within the volume. The physics behind this interaction relies on the magnetic stray field generated by the magnetic sample, which is detected by the tip through the magnetostatic interaction energy^12–15^. How this detection takes place corresponds to variations in the cantilever oscillation frequency due to force gradients on the magnetic tip generated by the vertical displacement of the cantilever’s end. Also, the magnetic tip should be close enough to get a clear image but not so close to prevent deformations on the sample magnetization^5^. By assuming that variations in tip height are small compared to the tip-sample distance, the problem of theoretically characterizing an MFM measurement reduces to calculating the magnetic force exerted over the sample or the tip. There are several reports in which the magnetic force is obtained for different geometrical and magnetic configurations between the tip and the sample by using distinct approximations^4,14,16–22^. In most of them, the calculation of the magnetostatic interaction energy between tip and sample is carried out on the tip volume, which requires knowing the tip’s magnetization and the stray field generated by the sample. The typical assumption is modeling the sample’s magnetization pattern as a periodically variable one^5^, which facilitates the calculation of the stray field when transforming into the Fourier space. These assumptions indeed allow defining the so-called tip transfer function (TTF)^23^, a quantity that relates the magnetic force (in the frequency domain) with the sample’s magnetization. By using the *monopole* approximation^4,15,22,24,25^, the TTF can be readily calculated providing an estimate of the MFM sensitivity^26^ for samples with periodic magnetic patterns.

However, when considering localized magnetic patterns such as magnetic solitons, calculations in Fourier space are less efficient and the theoretical analysis becomes more convenient to be performed in real space. A magnetic soliton is a localized magnetic texture characterized by a typical length scale.^27–29^. Examples of magnetic solitons are domain walls^27,30^ and magnetic skyrmions^31,32^, whose characteristic lengths are the domain wall width and the skyrmion diameter, respectively. Besides, magnetic solitons are keys for nanodevices such as nanosensors, nanotransistors, and nano oscillators, among others^31,33–36^. An MFM measurement of such magnetic configurations should be performed carefully, as the characteristic length could suffer variations produced by the interaction with the tip. These variations are significant compared to the ones caused by thermal fluctuations. Indeed, the latter are negligible since, otherwise, MFM measurements would not be possible. In this context, the monopole approximation and the use of the TTF (derived from a periodic magnetization pattern) to calculate the MFM sensitivity may no longer be sufficient. While several studies have applied different mathematical tools to the TTF approach to obtain theoretical MFM signals for skyrmions^37–41^, these reports focus exclusively on skyrmions and the tools used are tailored to this specific magnetic texture. Therefore, a comprehensive theoretical analysis that takes into account the influence of the tip on different texture properties and allows an accurate determination of the MFM sensitivity is essential for experiments with a broader range of magnetic solitons.

In this manuscript, we analytically investigate both the sensitivity of MFM in spatial resolution and the impact of MFM measurements on the size of magnetic solitons, such as DWs and skyrmions. We compare our results with previous experimental studies on MFM regarding DW stripes and with micromagnetic simulations of the skyrmion-tip system. Our results show strong agreement with both simulation and experimental data.

## Model

To study analytically and numerically both the sensitivity of MFM in spatial resolution and the effect of an MFM measurement on the sample’s magnetization, we consider the interaction energy between the tip and sample. Due the reciprocity principle^28^, the magnetostatic interaction energy can be calculated by two ways: by integrating the coupling between the sample dipolar field and tip magnetization in the tip volume space, or by integrating the coupling between the tip dipolar field and sample magnetization in the sample volume space, i.e., $E_{\text {int}}= -\mu _0\int _{\text {tip}}\mathbf{M}_{\text {t}}\cdot \mathbf{H}_{\text {s}} dV_{\text {t}}= -\mu _0\int _{\text {sample}}\mathbf{M}_{\text {s}}\cdot \mathbf{H}_{\text {t}} dV_{{\text {s}}}$, where $\mathbf{M}$ and $\mathbf{H}$ are, respectively, the magnetization field and the generated stray field. The sub indices ‘s’ and ‘t’ stand for *sample* and *tip*, respectively. The magnetic force is then obtained by applying gradient, $\mathbf{F} = -\mathbf {\nabla }E_{{\text {int}}}$. The MFM sensitivity can be obtained by comparing the vertical force component over tip due to magnetostatic interaction with thermal effects^5,26^, giving a minimum detectable MFM signal, $l_{\text {th}}$. In our model, the noise representing thermal effects can also account for other sources of fluctuations, such as gases present during the measurement. According to the Newton’s third law, this can be calculated by the vertical force component over the sample ($F_{s_z}$) such that $l_{\text {th}}$ can be obtained through:

1 $$\begin{aligned} \biggl ( \frac{\partial F_{\text {s}_z}}{\partial d_z} \biggl )^2 = \frac{4k k_\text {B} T B}{\omega _0 Q A^2}, \end{aligned}$$

where *k* is the cantilever spring constant, *T* is the temperature at which the measurement is carried out, *B* the bandwidth related to the thermal noise source, $\omega _0$ is the cantilever resonance frequency, *Q* the quality factor, *A* the mean square amplitude, and $k_\text {B}$ is the Boltzmann constant.

**Fig. 1:**
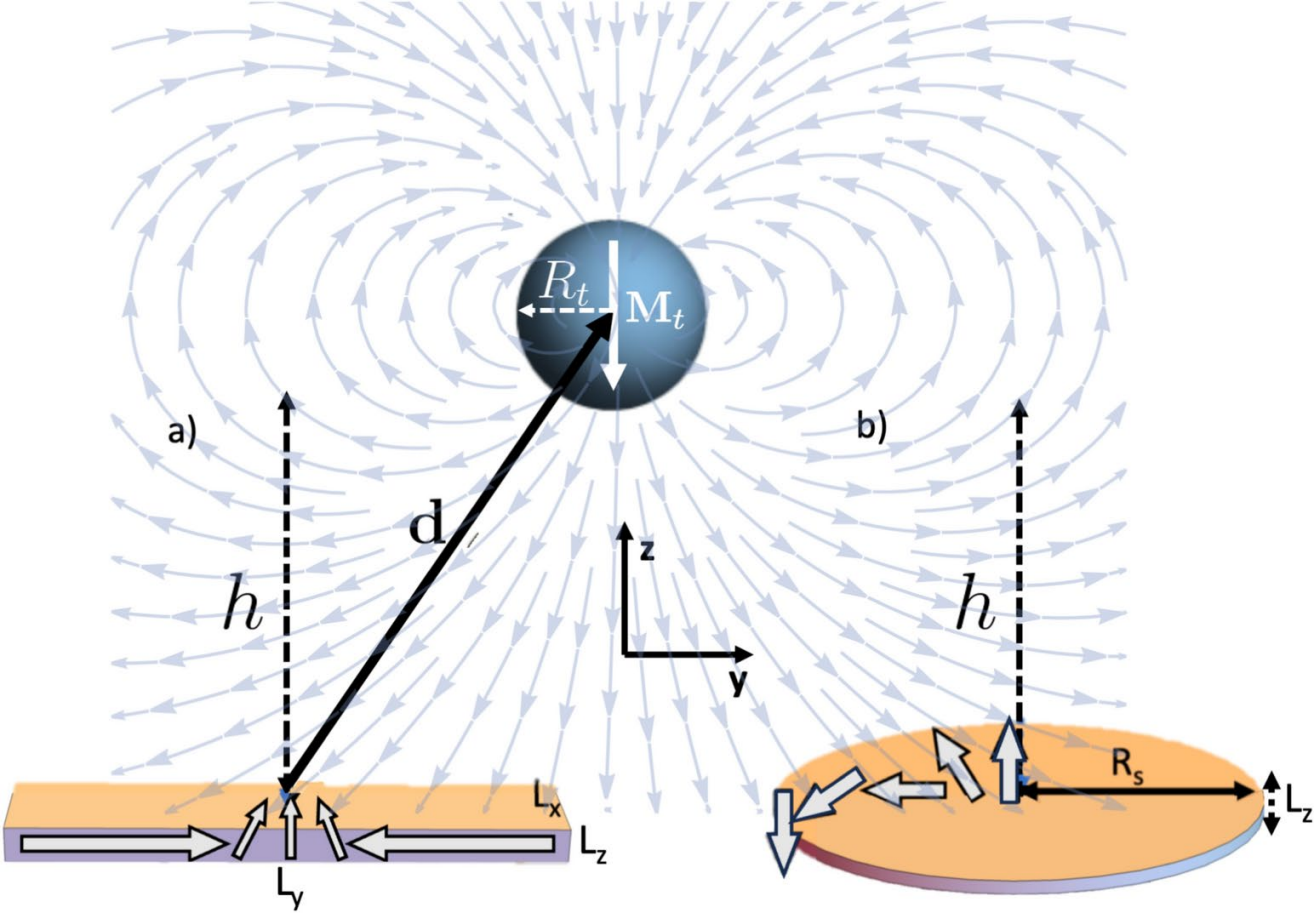
Schematic of a spherical MFM-like tip of radius $R_\text {t}$ and $\mathbf{M}_\text {t}=-M_\text {t}\hat{k}$ acting on (**a**) a rectangular thin stripe with dimensions $L_x,L_y,L_z$ and (**b**) a thin nanodisk with radius $R_\text {s}$ and thickness $L_\text {z}$. In both cases, the vertical distance of separation is $h = d_z-R_\text {t}$, and vector $\mathbf{d}$ is measured from the center of the magnetic texture to the tip center. The arrows in the background represent the stray field generated by the spherical tip, while the gray ones within the both samples depict their magnetization fields (DW and skyrmion), respectively.

The method to obtain the sensitivity in spatial resolution lies in the heart of Eq. (1), together with the implementation of any Ritz models to describe the magnetization of the sample, which is defined as a field vector that depends on Ritz parameters, $l_{\text {ch}}$, which are minimizable parameters characterizing the size of the magnetic texture in the sample. The texture is assumed to be non-deformable and motionless. The modeling accuracy depends on the Ritz model’s choice; in particular, a model with more minimizable parameters typically yields more accurate results^42^. For example, a Ritz model for a skyrmion could include parameters such as skyrmion radius, nucleus position, helicity, and time. However, for this study, it is sufficient to consider only the size of the soliton as a minimizable parameter. Therefore, for a given geometry and magnetic parameters of the sample and tip, the left hand side of Eq. (1) will be a function of $l_{\text {ch}}$ and the distance $\mathbf{d}$ between the tip and sample (see Fig. 1 and supplementary material). By solving numerically Eq. (1), $l_{\text {ch}}$ will correspond to the minimum detectable length, $l_{\text {th}}$, that the thermal noise allows to measure by MFM for a given cantilever parameters and temperature. Besides, the method also allows the analysis of the effects of a MFM measurement on the magnetization of the sample by minimizing the total magnetic energy of the sample, $E_{\text {s}}=E_\text {self}+ E_\text {int}$, as a function of the Ritz parameters $l_{\text {ch}}$. The term $E_{\text {self}}$ may consist of exchange, anisotropy, dipolar, Dzyaloshinskii–Moriya Interaction (DMI), and Zeeman energies. The interaction energy between the tip and sample is given by $E_{\text {int}}$. By denoting $l_{\text {ph}}$ as the physical value of the Ritz parameters that minimizes $E_{\text {s}}$, then we can compare the minimum detectable length allowed by thermal noise, $l_{\text {th}}$, with the corresponding physical length, $l_{\text {ph}}$. To get accurate measurements, at least $l_{\text {ph}}$ should be equal or greater than $l_{\text {th}}$, i.e., $l_{\text {th}}\le l_{\text {ph}}$. This is an important point, giving some insights into the magnetic texture’s possible size variation due to the magnetic tip’s presence in a typical MFM measurement.

## Results and analysis

We apply the model described above to characterize an MFM-like measurement of a domain wall in thin magnetic stripes and skyrmions in thin multilayered circular dots with out-of-plane magnetic anisotropy. The geometrical parameters for the sample and tip are depicted in Fig. 1. Here, we model the tip as a magnetic sphere of radius $R_t$, uniformly magnetized along the ± z-direction, i.e., $\mathbf {M_t} = \pm M_t\hat{k}$^43^ being $M_t$ the saturation magnetization of the tip. We consider a Cobalt tip, similar to Refs.^5,20^. The detail of the MFM tip and cantilever parameters are given in the “Methods” section. For these systems, the $z-$ component of the magnetic force applied over the sample can be written as

2 $$\begin{aligned} F_{\text {s}_z} = \pm \mu _0 M_{\text {s}} M_{\text {t}} \int _\text {sample} \hspace{-0.2cm}\mathbf{m}_{\text {s}}(\mathbf{r},l_\text {ch})\cdot \partial _z \mathbf{h}_{\text {t}}(\mathbf{r}-\mathbf{d})\, dV_{\text {s}} \end{aligned}$$

respect to a reference system fixed at the sample (see Supplementary material). In Eq. (2), $\mathbf{m}_\text {s}=\mathbf{m}_\text {s}/M_\text {s}$ is the normalized sample magnetization and $\partial _z\equiv \partial /\partial z$. Besides, we have defined the dimensionless stray field generated by the tip as $\mathbf{h}_\text {t}=\mathbf{H}_\text {t}/M_\text {t}$, and ± signs hold for tip magnetization in $\pm z$-direction. The use of a spherical tip offers several advantages. Different tip geometries can be effectively approximated as magnetic spheres with different radii of curvature. This simplification allows the use of closed analytical expressions for $\mathbf{h}_{\text {t}}$, preserving general effects while simplifying computations. Our approach is less restrictive than previous studies that modeled the magnetic tip as a simple magnetic dipole^38,44,45^. References^44,46^ have shown that a magnetic dipole can accurately represent some commercial tips by incorporating an effective magnetic moment that captures the tip-sample interaction strength. This is consistent with our use of the spherical approximation since a spherical tip can be considered an effective magnetic dipole, with its radius controlling the effective interaction volume. Details of the stray field generated by the spherical tip are provided in the Supplementary Information. For additional validation, we compared the MFM signal of the spherical tip model with that of a variable-radius tip, which mimics the shape of a real MFM tip from Ref.^40^ and found good agreement (see section V in the Supplementary Information). Most of commercial tips also share a similar shape^1,47^.

In the following, we analyze the sensitivity in spatial resolution and the effect of MFM measurements over DWs and Skyrmions. The details of the sample magnetic parameters and ansatz used for both textures can be found in the “Methods” section. Regarding the energy contributions, for the DW case, we included the exchange, anisotropy, and self-dipolar energy, while for the skyrmions sample, we included the previous magnetic energies together with the DMI energy. For both textures, we approximate the self-dipolar energy as an easy-in-plane anisotropy which can be accounted using an effective perpendicular magnetic anisotropy constant $K_{\text {eff}}=K_\text {u}-\mu _0M_\text {s}^2/2$, which is valid for a thin magnetic sample^48^.

To find $l_{\text {th}}$, Eq. (2) must be inserted into Eq. (1) and solve it numerically. For the DW case, we calculate Eq. (2) using Cartesian coordinates, i.e.:

3 $$\begin{aligned} F_{z} (\Delta ) & = \pm \mu _{0} M_{{\text{s}}} M_{{\text{t}}} R_{t}^{3} L_{x} L_{z} [\int_{{ - \infty }}^{\infty } {\frac{{m_{y} (y,\Delta )(y^{2} - 4d_{z}^{2} )}}{{(y^{2} + d_{z}^{2} )^{{7/2}} }}} ydy \\ & \quad + \int_{{ - \infty }}^{\infty } {\frac{{d_{z} m_{z} (y,\Delta )(2d_{z}^{2} - 3y^{2} )}}{{(y^{2} + d_{z}^{2} )^{{7/2}} }}} dy], \\ \end{aligned}$$

while for the skyrmion case it is convenient to use cylindrical coordinates:

4 $$\begin{aligned} F_{z} (r_{{\text{s}}} ) & = \pm 2\pi \mu _{0} M_{{\text{s}}} M_{{\text{t}}} R_{{\text{t}}}^{3} \\ & \quad \times \left[\int_{0}^{{L_{z} }} {\int_{0}^{{R_{{\text{s}}} }} {\frac{{\rho ^{2} {\mkern 1mu} d\rho {\mkern 1mu} dz{\mkern 1mu} (\rho ^{2} - 4(z - d_{z} )^{2} ){\mkern 1mu} m_{\rho } (\rho ,r_{{\text{s}}} )}}{{(\rho ^{2} + (z - d_{z} )^{2} )^{{7/2}} }}} } \right. \\ & \left. \quad + \int_{0}^{{L_{z} }} {\int_{0}^{{R_{{\text{s}}} }} {\frac{{\rho {\mkern 1mu} d\rho {\mkern 1mu} dz\;(z - d_{z} ){\mkern 1mu} (3\rho ^{2} - 2(z - d_{z} )^{2} ){\mkern 1mu} m_{z} (\rho ,r_{{\text{s}}} )}}{{(\rho ^{2} + (z - d_{z} )^{2} )^{{7/2}} }}} } \right], \\ \end{aligned}$$

where we have considered the tip located at a vertical distance $d_z$ respect to the sample. The details of the calculations of these equations are given in Section II of the Supplementary Material. Besides, for the selected magnetic textures, the Ritz minimizable parameters ($l_{\text {ph}}$) correspond to the domain wall width, $\Delta$, and skyrmion diameter $2 \, r_\text {s}$.

**Fig. 2:**
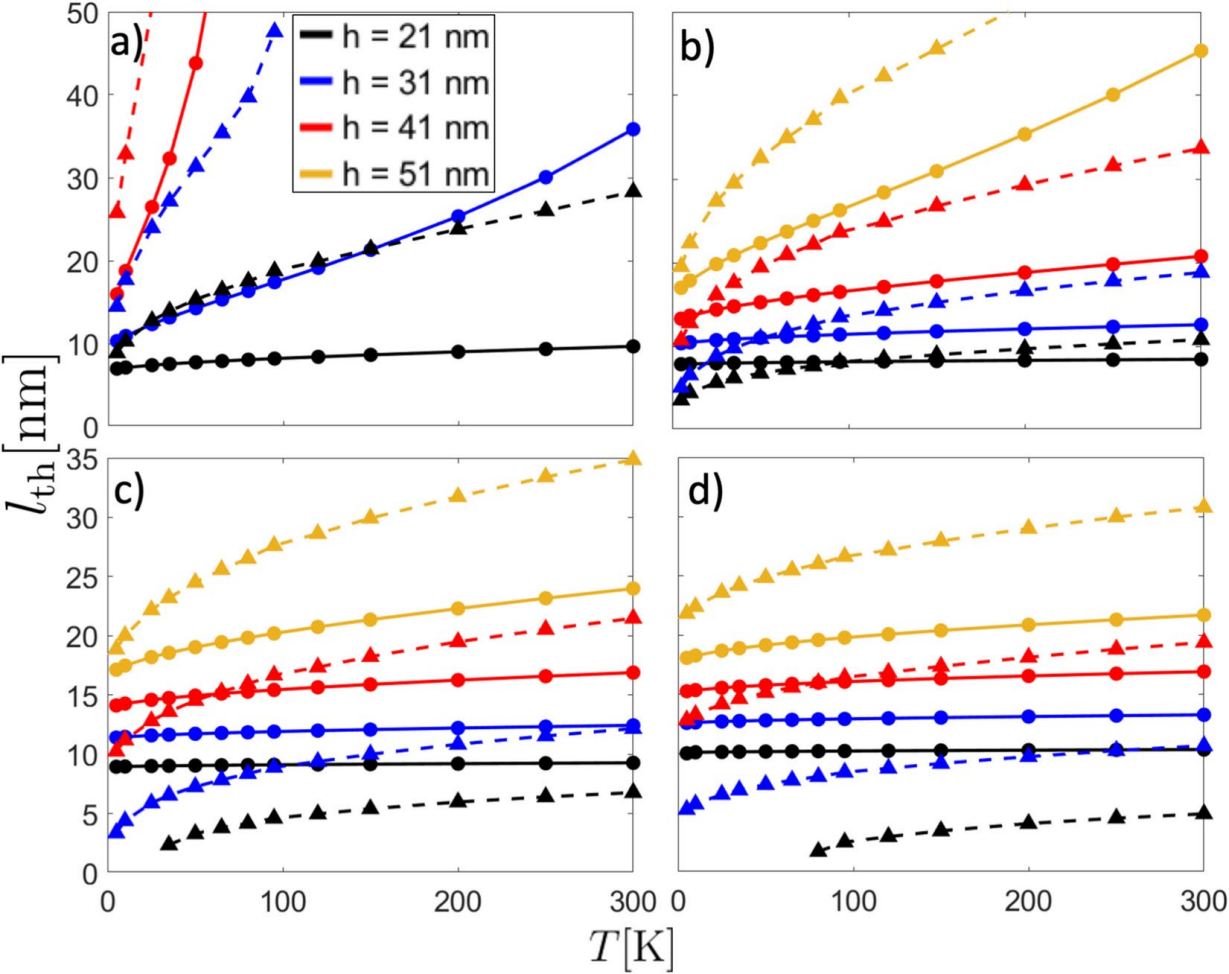
Minimum detectable MFM signal allowed by the thermal noise, $l_{\text {th}}$, as a function of temperature for different distances of separation and (**a**) $R_\text {t} = 5$ nm, (**b**) $R_\text {t} = 10$ nm, (**c**) $R_\text {t} = 15$ nm and (**d**) $R_\text {t} = 20$ nm. Filled circles with full lines correspond to DW, while filled triangles with dashed lines hold for skyrmion sample.

In Fig. 2, we show $l_\text {th}$ as a function of temperature for different tip radii and distances of separation *h* for both textures. Circular markers with full lines correspond to the domain wall case, while triangular markers with dashed lines hold for the skyrmion sample. Note that since Eq. (1) is quadratic in the magnetic force, so that the sign choice in the tip magnetization does not produce any effect on the minimum detectable signal $l_\text {th}$ of the textures. As expected, $l_\text {th}$ increases with both the temperature and distance of separation since thermal fluctuations could induce oscillations of the cantilever and because the magnetostatic interaction decreases with the tip-sample distance, too. In general, for a given temperature, $l_\text {th}$ is larger for the skyrmion case compared to the DW sample, except for the most interacting case ($h=21$ nm) and for tip radii 15 and 20 nm, where skyrmions of length close to 5 nm could be detected at room temperature. One can notice that for the most interacting case, there is not a valid solution for the skyrmion system for temperatures below $T \lessapprox 38$ K and $T \lessapprox 80$ K when $R_\text {t} = 15$ and 20 nm, respectively, since Eq. (1) does not accept valid solutions for such a set of parameters. Note that in all the cases $l_{\text {th}}$ varies effectively with the temperature.

**Fig. 3:**
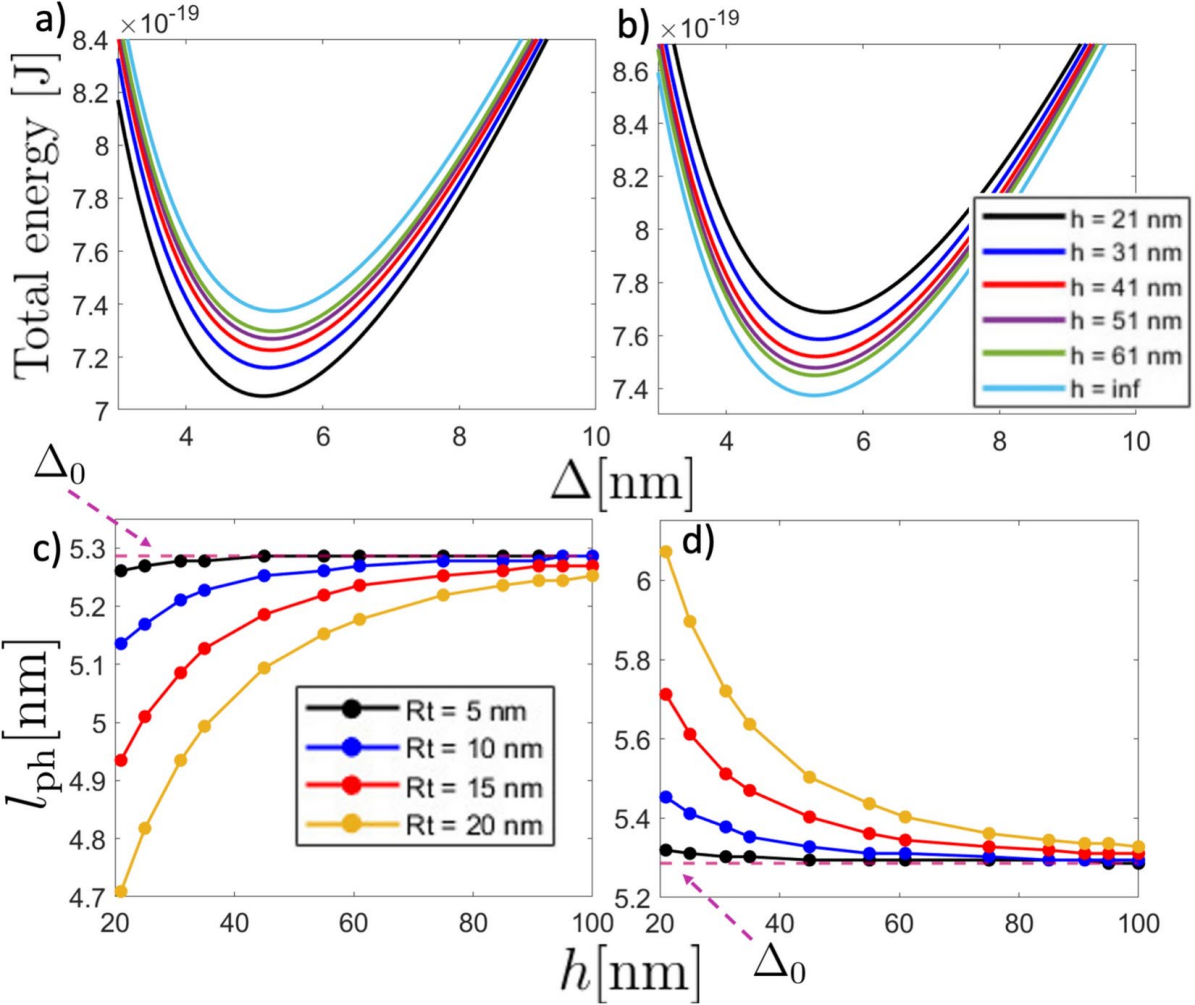
(Upper panel) Total energy of the magnetic stripe as a function of the DW width $\Delta$ for different distances of separation with $R_\text {t}=10$ nm and considering (**a**) $\mathbf{m}_t = -\hat{k}$ and (**b**) $\mathbf{m}_t = +\hat{k}$. Note the difference in the horizontal scales. (Lower panel) Physical length $l_{\text {ph}}$ as a function of the distance of separation for different tip radii and considering (**c**) $\mathbf{m}_t = -\hat{k}$ and d) $\mathbf{m}_t = +\hat{k}$. The pink dashed line stands for the specific DW width $\Delta _0$ in the absence of the magnetic tip.

**Fig. 4:**
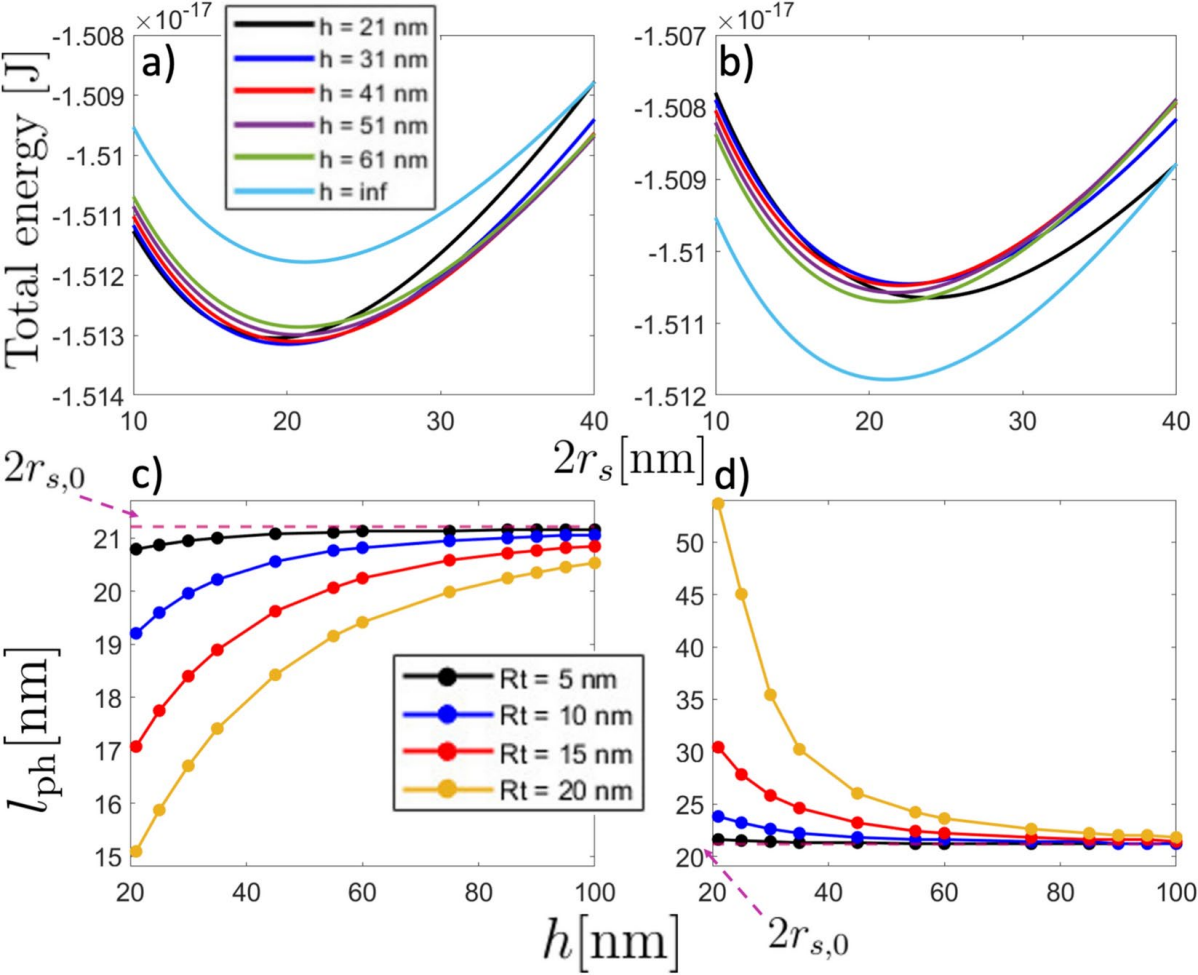
(Upper panel) Total energy of the magnetic nanodot hosting a skyrmion as a function of the skyrmion diameter $2r_\text {s}$ for different distances of separation with $R_\text {t}=10$ nm and considering (**a**) $\mathbf{m}_t = -\hat{k}$ and (**b**) $\mathbf{m}_t = +\hat{k}$. (Lower panel) Physical length $l_{\text {ph}}$ as a function of the distance of separation for different tip radii and considering (**c**) $\mathbf{m}_t = -\hat{k}$ and (**d**) $\mathbf{m}_t = +\hat{k}$. The pink dashed line pointed by the dashed arrow stands for the specific skyrmion diameter $2r_\text {s,0}=21.1$ nm in the absence of the magnetic tip.

Now, we calculate the physical length $l_{\text {ph}}$ that the magnetic textures acquire in the presence of the tip, which corresponds to the characteristic length that minimizes the total energy. In Figs. 3 and 4 we show both the total energy and $l_{\text {ph}}$ for DW and skyrmion case, respectively. In these figures, panels (a) and (b) show the total energy as a function of the characteristic texture length ($\Delta$ for the DW and $2r_\text {s}$ for the Skyrmion) for different distances of separation *h*, fixing $d_y=0$ and $R_\text {t}= 10$ nm, and considering tip magnetization direction $\mathbf {m_\text {t}} = -\hat{k}$ and $+\hat{k}$, respectively. Additionally, panels c) and d) show the physical texture length as a function of *h* for different tip radius when $\mathbf {m_\text {t}} = -\hat{k}$ and $+\hat{k}$, respectively. As expected, $l_{\text {ph}}$ converges to the non-interacting case, i.e., $\Delta _0$ for DW and $2r_\text {s,0}$ for skyrmion, as the distance of separation becomes larger. Regarding the DW sample, the physical DW width slightly deviates from its non-interacting value $\Delta _0 \approx 5.3$ nm when the tip points opposite to the DW magnetization direction. Furthermore, the influence of the tip on the DW size becomes more prominent as the tip radius increases and when the tip and DW magnetizations align. However, in the last case, the DW size variation is not significant as it produces small changes in DW width. To compare $l_\text {th}$ with $l_\text {ph}$, we can use Figs. 2 and 3c–d. By assuming that $l_{\text {ph}}$ holds for a relatively wide range of temperatures $T\lessapprox 300$ K, we can see that the condition $l_{\text {th}}\le l_{\text {ph}}$ never occurs. Indeed, from Fig. 2, one can notice that by using a tip with radius $R_\text {t} = 20$ nm with a distance of separation $h=21$ nm at room temperature, the minimal detectable signal $l_{\text {th}}$ is about 10 nm. Additionally, from Fig. 3c, when the tip points opposite to the DW magnetization center, the physical DW width $l_{\text {ph}}$ is about 4.7 nm. In contrast, $l_{\text {ph}}$ is about 6.1 nm if the tip aligns with the DW magnetization center. Therefore, no matter the magnetization direction of the tip, the measurement will always be overestimated in this particular case.

On the other hand, for the skyrmion sample, the skyrmion diameter can be strongly changed respect to its non-interacting value $2r_{s,0 }\approx 21.1$ nm. Recall $r_{s,0}$ strongly depends on the sample parameters and the eventual external magnetic field, resulting in sizes ranging from dozens of nanometers to hundreds of them^49,50^. Note that, similar to the DW case, major size variations of skyrmion diameter occur when the tip’s magnetization aligns with the skyrmion core direction ($+\hat{k}$). Here, the alignment between the magnetostatic field lines of tip and skyrmion core increases the radius of such a magnetic texture. This implies that when the magnetization of the tip is aligned to the skyrmion core magnetization and the tip approaches the sample, the skyrmion tends to expand its radius, potentially transitioning into a quasi-uniform magnetic state^49,51^ (see Fig. 4d). Note that the stray field from the tip acts as an external magnetic field, directly affecting the skyrmion radius. Figures 2 and 4c, d evidence that $l_{\text {th}}$ is, at least, similar to $l_{\text {ph}}$. For instance, for the case $R_\text {t}=20$ nm evaluated at 300 K and $h=21$ nm, $l_{\text {th}}\approx 5.8$ nm (Fig. 2d). For the same parameters, $l_{\text {ph}}\approx 15.2$ nm for the tip opposite to the skyrmion core (Fig. 4c) and $l_{\text {ph}}\approx 53.7$ nm for the tip parallel to the skyrmion’s core magnetization (Fig. 4d). Therefore, in this specific case, the skyrmion size will appear about $28\%$ smaller (from its non-interacting value) when the tip is opposing the magnetization in the center of the skyrmion core and about $60\%$ larger when it is parallel to it. Unlike the domain wall texture, here we can anticipate that MFM measurements will yield reliable results because the condition $l_{\text {th}}<l_{\text {ph}}$ is fulfilled.

For completeness, we have compared the model for the DW stripe sample with published experimental results available in the literature. Although typical experiments are conducted under conditions beyond the model presented here, we can still make some estimations for realistic measurements. For instance, in references^52–54^, the authors show that there is a notorious difference in the domain wall width when changing the DW magnetization direction, which in turn is equivalent to changing the tip magnetization in our model. The two distinct modes are called *attractive* (tip and sample magnetization pointing in the same direction) and *repulsive* (tip and sample magnetization pointing in the opposite direction). Additionally, most measurements are carried out in remanence, which our model cannot include as it implies a given magnetic history through the hysteresis loop. For DWs corresponding to metastable states, this is a standard routine to get DWs to be imaged. However, we can crudely simulate the state of remanence in the model by considering an external applied magnetic field since it stabilizes the DW, which could be considered to play the same role as the remanent state when performing MFM measurements. . We first show that an external magnetic field of a few hundred mT allows us to enhance the DW width, reaching relatively similar values as the experimental reports. Indeed, for a sample with the same magnetic parameters as used in the DW case above, and considering $L_x = 500$ nm, $L_y= 1000$ nm, and $L_z=50$ nm, we get $\Delta _0\approx 48.1$ nm when applying a magnetic field of $\mathbf{B}=340 \hat{k}$ mT, as shown in Fig. A4c of the Supplementary Material Sec. IV. In such section, we also show the effect of the tip on $l_{\text {ph}}$ as a function of *h* and the tip radius $R_\text {t}$ while the external magnetic field is applied. We have considered the two distinct configurations mentioned above, i.e., for repulsive configuration in Figs. A5a and A5c, and attractive configuration Figs. A5b and A5d, showing a variation in the DW width between both modes that qualitatively agrees with the experimental results in Ref.^53^.

**Fig. 5:**
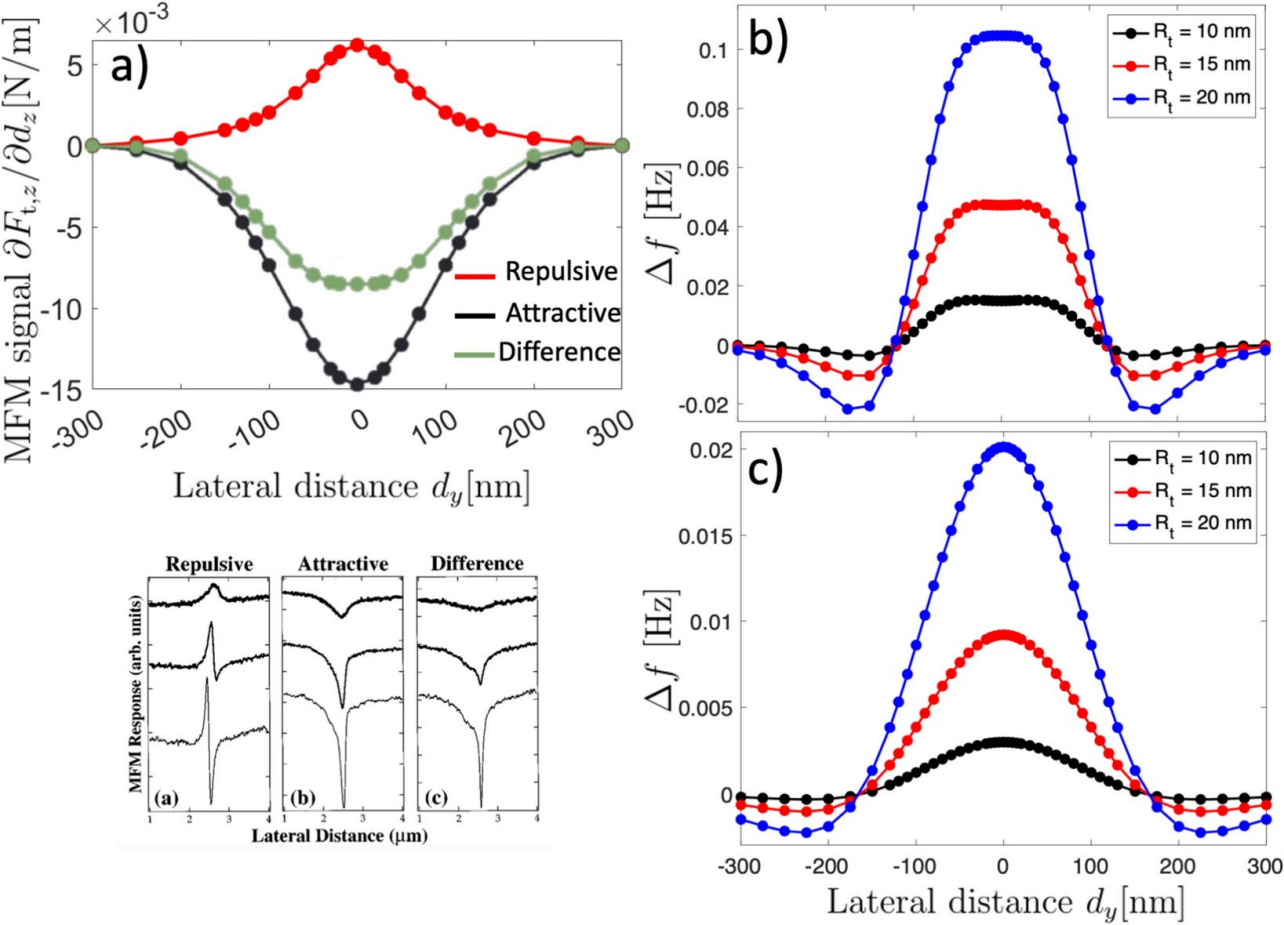
(**a**) Domain walls: theoretical force gradient over the tip as a function of the lateral distance $d_y$ for repulsive, attractive, and its corresponding difference between the attractive and the inverted repulsive profiles. The inset (lower panel) corresponds to experimental results from Fig. 3 in Ref.^54^. (**b**) and (**c**) Skyrmions: frequency shift as calculated from Refs.^40,55,56^, as a function of the lateral distance $d_y$ for (**b**) $h=100$ nm and (**c**) $h=200$ nm.

Besides, we can go further by performing calculations of the MFM response, maintaining the vertical sample-tip separation fixed while varying the lateral distance $d_y$ along the nanostripe axis to estimate the effect of a horizontal scan of the tip at a fixed height over the DW. We assumed that the DW is pinned at $d_y=0$, so there are no changes in the position of the wall, but only its width changes (see Figs. A4a and A4b in Supplementary material). Figure 5a shows the MFM signal, defined as $\partial F_{\text {t},z}/\partial d_z$^53,54^, as a function of the lateral position $d_y$, being $F_{\text {t},z}$ the $z-$component of the magnetic force over the tip, in the repulsive (red line) and attractive modes (black line). This force gradient is basically the same as the response obtained in experimental MFM profiles, as evidenced in Figure 3 of Ref.^53^ and Figures 2 and 3 of Ref.^54^. Here, we calculated the magnetic force using Eq. (2) replacing $l_\text {ch}$ by the corresponding $l_\text {ph}$ values and after applying an overall minus sign due to Newton’s third law. As can be seen, there is a marked difference in the force gradients between the repulsive and attractive modes. In Fig. 5a, we also quantify such a difference (green line) by subtracting the inverted repulsive profile from the attractive one. Importantly, these are typical profiles as obtained from MFM measurements. Indeed, Figure 3 in Ref.^54^ shows the same behavior for the scanning of a $180^{\circ }$ DW. For comparison, we explicitly included these experimental results as an inset in Fig. 5a. It can be seen that the model results have the same behavior as the experimental results for the attractive and repulsive modes, including their differences. Nevertheless, the experimental results (inset) show greater deformations of the DW structure as long the sample-tip separation becomes smaller, evidenced by noticeable asymmetries in the MFM profile, behavior that the model can not cast as it does not include other types of deformations of the DW apart of their sizes. It is important to point out that, in addition to thermal noise, typical measurements are also limited by other noise sources attributed to the presence of lasers, amplifiers, vibrations, or tip damping. Therefore, the presence of an external magnetic field in our model intends to qualitatively mimic most of the noise source contributions.

**Fig. 6:**
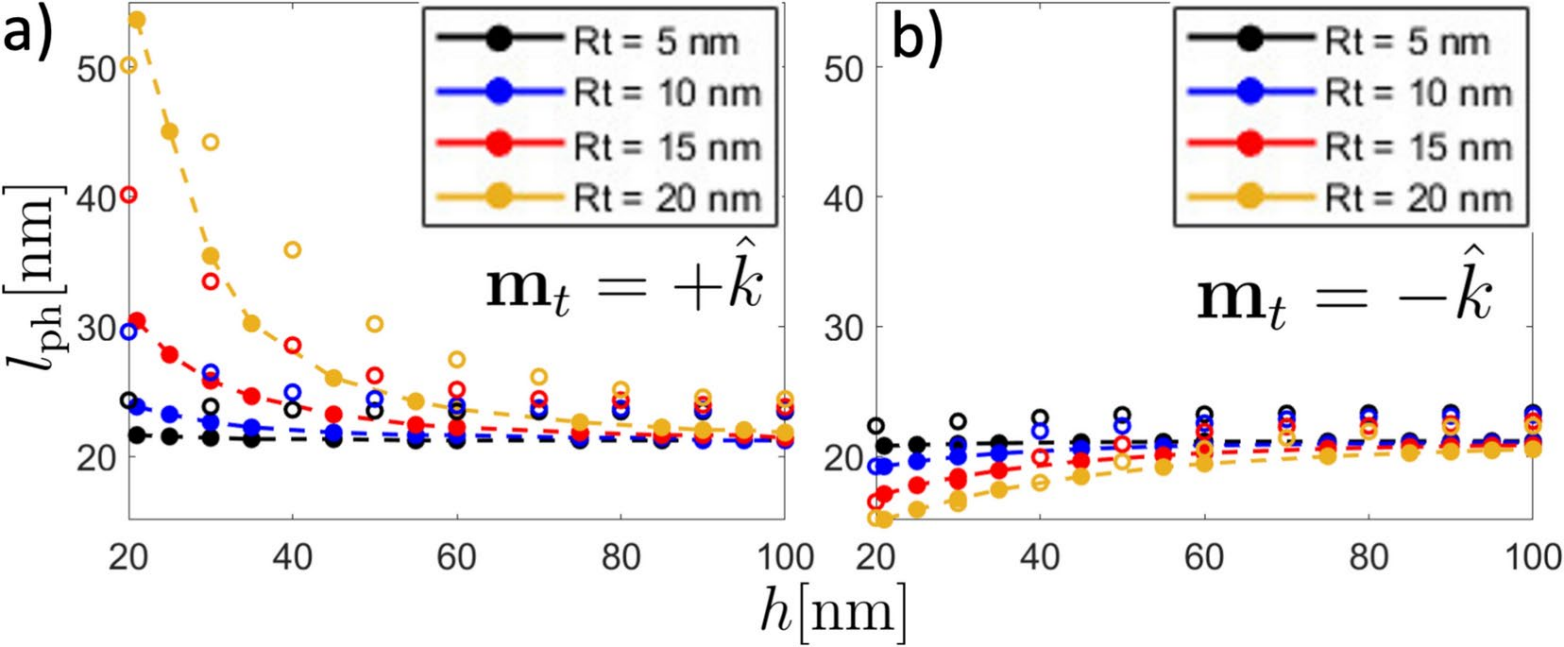
Physical skyrmion diameter $l_{\text {ph}}$ as a function of the distance of separation *h* for different tip radius $R_\text {t}= 5$ nm (black), $R_\text {t}= 10$ nm (blue), $R_\text {t}= 15$ nm (red), and $R_\text {t}= 20$ nm (yellow). Filled symbols with dashed lines correspond to the results of the theoretical method, while open symbols correspond to micromagnetic simulations. Panel a) stands for the *atractive* mode (tip magnetization pointing in the same direction of the skyrmion core), while b) stands for the *repulsive* one (tip magnetization pointing opposite to the direction of the skyrmion core).

Concerning the Skyrmion textures, there are some reports in which such textures are imaged and nucleated by MFM^10,37–41,57,58^. Typical MFM measurements focus on the frequency shift, $\Delta f$, during a horizontal sweep. In Fig. 5b, c, we present the frequency shift calculated as described in Refs.^40,55,56^ for various tip radii. Specifically, Fig. 5b represents a tip-sample distance of $h = 100$ nm, while Fig. 5c corresponds to $h = 200$ nm. The frequency shift obtained qualitatively matches the results reported in Ref.^40^, providing a good approximation to experimental findings. Nevertheless, none of them pay special attention to their possible deformations. Therefore, we have compared the predicted physical skyrmion diameter in the presence of the tip with micromagnetic simulations (see details in the Micromagnetic simulations subsection in the “Methods” section). In Fig. 6, we show both simulations (open markers) and model results (filled markers with dashed lines) for the physical skyrmion diameter as a function of the separation distance between the tip and the sample hosting a skyrmion with positive polarity. Figure 6a corresponds to the case in which both the tip and the skyrmion core point in the same direction (+$\hat{k}$), while Fig. 6b depicts the opposite case, in which the tip has a contrary magnetization to the skyrmion polarity. As can be seen, in both attractive and repulsive cases there is a good agreement between analytical calculations and micromagnetic simulations. For the non-interacting state, which can be assumed for a large *h*, the micromagnetic simulations and calculations differ by approximately 3 nm. This discrepancy can be atributed to the assumption of the self-dipolar energy as an anisotropy constant $-\mu _0M_\text {s}^2/2$, where we considered a demagnetizing factor $N_{zz}=1$. Due to the size of the nanodisk, a tiny reduction in $N_{\text {zz}}$ should be considered, which implies a small increase in the effective anisotropy constant $K_{\text {eff}}$ and, consequently, an increase of the Skyrmion diameter. On the other hand, for the attractive case (see Fig. 6a), the micromagnetic simulations and calculations show a more pronounced deviation of the physical length for small values of *h*. Nevertheless, in the case $R_\text {t}=20$ nm, the calculations deviates more significantly compared to the simulated case for $h<30$ nm (see Fig. 6a). This behavior can be attributed to the model assuming a rigid tip’s magnetization, whereas in the simulations some deviations of the magnetization are expected. Such deviations reduce the magnetic charges in the spherical tip, thereby reducing the stray field. Thus, the interaction between the Skyrmion and the tip is more significant in the calculations than in the simulations.

## Conclusions

In this work, we have developed a method to theoretically characterize the size variation of the magnetic texture in an MFM measurement by considering both thermal fluctuations and the force between the magnetic tip and the sample magnetization. The magnetization texture can be imaged when the physical characteristic length of the texture in the presence of the MFM tip ($l_{\text {ph}}$) is equal or greater than the thermal noise limited resolution ($l_{\text {th}}$) of the instrument. However, the MFM tip will still modify the texture. A measure for the influence of the tip is the difference between the observed length of the texture and the theoretical length if the tip were infinitely far away $\vert l_{\text {ph}} - l_\text {ph} (h\rightarrow \infty ) \vert$. This value depends on the tip magnetization direction through $l_{\text {ph}}$. As a result, one will overestimate the characteristic length from the MFM image when $l_\text {ph} \le l_\text {th}$ whereas $l_\text {ph}$ may still be smaller than $l_\text {ph} (h\rightarrow \infty )$.

We have shown that the spherical tip model is sufficient to capture most of the physical effects in tip-sample interactions. In MFM measurements, the interaction is primarily characterized by an effective interaction volume corresponding to the part of the tip closest to the sample. By adjusting the radius of the spherical tip, this model effectively represents the interaction volume. In addition, our approach is quite general and requires only an estimate of the magnetization profile of the sample. Next to skyrmions these include magnetic vortices, helical states and chiral domain walls. It can be obtained by using micromagnetic simulations or numerically solving the corresponding Euler-Lagrange equations.. This framework allows for accurate modeling and can be adapted to more complex Ritz models with additional representative and minimizable parameters, such as helicity (which might serve to distinguish Néel and Bloch skyrmions) or core displacements, which would improve the model and allow it to reach more realistic results.

When applied to the case of a domain wall, our model shows qualitative agreement with published MFM measurements. By adjusting parameters such as the height of separation, the tip radius, and the inclusion of an external magnetic field, we found a good qualitative agreement between our model and experimental results in both attractive and repulsive modes. The model does not only predict the correct wall width, but also the change in wall profile for attractive or repulsive wall-tip interaction.

To further assess our model, we compared its prediction to micromagnetic simulations for a tip-skyrmion system, obtaining a maximum difference of 6 nm for the skyrmion diameter. In addition, our model shows a good qualitative agreement with experimental measurements of the frequency shift $\Delta f$ for this system.

It is important to consider the concept of temperature carefully, as it effectively incorporates any noise source. In the proposed model, the thermal noise-limited resolution is obtained by comparing the vertical force component over the tip, due to magnetostatic interaction, with thermal effects. Thus, the precision of the lower limit for $l_{ph}$ on MFM is determined by the applicability of Eq. 1, while the upper limit is constrained by the maximum lateral distance in MFM scanning.

The proposed method is general and can be applied to different magnetic textures with a characteristic length, where the magnetization does not vary significantly throughout the sample volume. The magnetic sample should be well described by a magnetization vector field ansatz and a minimizable magnetic energy functional that depends on the texture’s characteristic length. Thus, this method is suitable for the experimental measurement of magnetic textures that can be well described by a magnetization ansatz. Examples include domain walls, skyrmions, skyrmioniums, domain wall skyrmions, Bloch lines, and magnetic vortices^51,59–62^. However, if the underlying magnetic structure is unknown, it is first necessary to determine an appropriate magnetization ansatz that accurately describes it, which can be done by employing micromagnetic simulations or solving the corresponding Euler–Lagrange equations.

Our approach is universal, as it can describe different materials’ tips by adjusting the spherical tip’s parameters. Possible applications involving magnetic textures are thought to be functionalized or manipulated as single magnetic solitons whose magnetization structure is as unperturbed as possible, especially in the absence of magnetic fields. In this sense, it becomes essential to know the physical length of magnetic textures that could configure a necessary ingredient for future applications and, at the same time, avoid considerable size variations when exposed to MFM measurements. Hence, our findings should be taken into account when measuring characteristic lengths of different magnetic textures so that the MFM measurements are not overestimated due to the soliton size variation induced by the presence of the magnetic tip. Our model reproduces both experimental measurements and micromagnetic simulations well. However, to achieve greater agreements, it is necessary to make the model more complex by incorporating more degrees of freedom into the Ritz model. This is left for prospective studies as we believe it deserves to be addressed deeply in future works. As future work it would be interesting to complement the model, including non only thermal effects but also analyzing the effect of other noise such as laser noise, amplifier noise, and vibrations, separately.

## Supplementary material

In the Supplementary material, we provide major details of the calculation of the magnetostatic field generated by the spherical tip, interaction energy, coordinates transformation, total micromagnetic energy, the numerical evaluation of $l_{\text {th}}$ and the effect of an additional external magnetic field on $l_\text {ph}$ for the DW sample in the presence of the tip. Finally, we compare the MFM signal of the spherical modeled tip with the signal of a more complex modeled tip with variable radius considered in Ref.^40^.

## Methods

### System magnetic parameters and texture ansatz

For the DW case, we consider a Permalloy (Py) nanostripe of size $L_x =15$ nm, $L_z=5$ nm, and $L_y\gg L_x,L_z$, with $M_\text {s} = 8.6\times 10^5$ A/m, $A_{\text {ex}}=1.3\times 10^{-11}$ J/m, and $K_\text {u}=0$^63^. This choice of DW corresponds to a one-dimensional domain wall lying along the $y-$axis and whose magnetization field stands at the $y-z$ plane, as represented in Fig. 1a, and given by $\mathbf{m}_\text {s} = m_y(y,\Delta )\hat{y} \,+ \, m_z(y,\Delta )\hat{z}$, with the Ritz function^28^ $m_y(y,\Delta ) = \tanh \left( \frac{y - y_0}{\Delta }\right)$, where $y_0$ corresponds to the position of DW center, and $\Delta$ is the DW width. In the absence of interaction between the tip and sample, the DW width becomes $\Delta _0=\sqrt{A_\text {ex}/{|K_\text {eff}|}}$.

On the other hand, for the skyrmion case, we consider a magnetic skyrmion hosted in a cylindrical Pt/Co/Pt thin nanodot as represented in Fig. 1b. The magnetic sample is characterized by: $M_\text {s} = 580$ kA/m, $L_z=1$ nm, nanodot radius $R_\text {s}=100$ nm, $A_{\text {ex}} = 15 \times 10^{-12}$ J/m, $K_\text {u} = 0.7 \times 10^{6}$ J/m^3^ and the interfacial Dzyaloshinskii–Moriya (DM) parameter $D = 3 \times 10^{-3}$ J/m^2^^50^. To describe the magnetization profile of skyrmions hosted in thin films, we use a normalized magnetization vector $\mathbf{m}_\text {s}= \, m_{\rho }(\rho ,r_\text {s}) \hat{\rho } + m_z(\rho ,r_\text {s}) \hat{z}$, with the ansatz^50,51,64^ $m_z(\rho ,r_\text {s}) = -P \cos [2 \arctan (f(\rho ,r_\text {s}))],$, where $P = \pm 1$ is the skyrmion polarity, which we fix equal to $P=+1$, $f(\rho ,r_\text {s}) = (r_\text {s}/\rho ) \exp \left( \frac{\xi }{l_{ex}} (r_\text {s} - \rho ) \right)$, with $l_{ex} = \sqrt{2A_{ex}/\mu _0 M_\text {s}^2}$, $\xi ^2 = C - 1$ and $C = 2K_\text {u}/\mu _0 M_\text {s}^2$. Here, $r_\text {s}$ is the skyrmion radius.

Finally, for the tip and cantilever, we consider the parameters given in Refs.^5,20,27^, i.e. a Cobalt spherical tip with saturation magnetization $M_\text {t}=1.44 \times 10^6 \text {A/m}$ attached to a cantilever with $Q = 3000$, $k = 2.8 \text {N/m}$, $\omega _0 = 75 \times 10^3 \, \text {Hz}$, $B = 200 \, \text {Hz}$, $A = 15$ nm.

### Micromagnetic simulations

We performed micromagnetic simulation for the skyrmion-tip sample using the in-house CUDA native micromagnetic solver, PETASPIN, which numerically integrates the Landau–Lifshitz–Gilbert (LLG) equation by applying the time solver scheme Adams–Bashforth^65^. For the simulations, we used the same geometrical and magnetic parameters of the skyrmion sample in the presence of a spherical tip and to carry out the analysis, we perform two set of simulations, one to calculate the magnetostatic field due to the MFM spherical tip, and another one to calculate the effect of the magnetostatic field due to the tip on the skyrmion size. For the former, we consider the spherical tip placed on top of the sample and centered respect to disk center, such that we are able to compute the magnetostatic field outside the tip for a distance ranging from 0 to 100 nm. The tip is considered with a uniform magnetization along either the positive or negative out-of-plane direction z-axis, and we use a cubic discretization cell of $1\times 1 \times 1$ nm^3^. For the latter, we perform simulations of the disk, with initial state as a Néel skyrmion placed in the middle of the disk. From the first set of simulations, we extract the magnetostatic field at the specific distance from the tip and we apply this non-uniform field as an external field to the skyrmion-based disk. The skyrmion radius was calculated as the radius of the area enclosed within the core skyrmion region.

## Supplementary Information

Supplementary Information.

## Data Availability

The data that support the findings of this study are available from the corresponding author upon reasonable request.

## References

[1] Kazakova, O. et al. Frontiers of magnetic force microscopy. *J. Appl. Phys.* **125, **060901 (2019).

[2] Koblischka, M. R. & Hartmann, U. Recent advances in magnetic force microscopy. *Ultramicroscopy* **97**, 103–112 (2003). 12801662 10.1016/S0304-3991(03)00034-2

[3] Vokoun, D., Samal, S. & Stachiv, I. Magnetic force microscopy in physics and biomedical applications. *Magnetochemistry* **8**, 42 (2022).

[4] Abelmann, L. Magnetic force microscopy. In *Encyclopedia of Spectroscopy and Spectrometry*, 675–684 (Elsevier, 2017). 10.1016/b978-0-12-803224-4.00029-7.

[5] Abelmann, L., van den Bos, A. & Lodder, C. Towards higher resolution in magnetic force microscopy. In *Magnetic Microscopy of Nanostructures, 2005th Edition* 254–283 (Springer, 2005).

[6] Manke, I. et al. Three-dimensional imaging of magnetic domains. *Nat. Commun.* **1**, 125 (2010). 21119638 10.1038/ncomms1125

[7] Szmaja, W. Recent developments in the imaging of magnetic domains. *Adv. Imaging Electron Phys.* **141**, 175–256 (2006).

[8] Birch, M. et al. Real-space imaging of confined magnetic skyrmion tubes. *Nat. Commun.* **11**, 1726 (2020). 32265449 10.1038/s41467-020-15474-8 PMC7138844

[9] Boulle, O. et al. Room-temperature chiral magnetic skyrmions in ultrathin magnetic nanostructures. *Nat. Nanotechnol.* **11**, 449–454 (2016). 26809057 10.1038/nnano.2015.315

[10] Meng, K.-Y. et al. Observation of nanoscale skyrmions in sriro3/srruo3 bilayers. *Nano Lett.* **19**, 3169–3175 (2019). 30935207 10.1021/acs.nanolett.9b00596

[11] Okuno, T., Shigeto, K., Ono, T., Mibu, K. & Shinjo, T. MFM study of magnetic vortex cores in circular permalloy dots: Behavior in external field. *J. Magn. Magn. Mater.* **240**, 1–6 (2002).

[12] Rugar, D. et al. Magnetic force microscopy: General principles and application to longitudinal recording media. *J. Appl. Phys.* **68**, 1169–1183 (1990).

[13] Schönenberger, C. & Alvarado, S. Understanding magnetic force microscopy. *Z. Phys. B Condens. Matter* **80**, 373–383 (1990).

[14] Porthun, S., Abelmann, L. & Lodder, C. Magnetic force microscopy of thin film media for high density magnetic recording. *J. Magn. Magn. Mater.* **182**, 238–273 (1998).

[15] Hartmann, U. Magnetic force microscopy. *Annu. Rev. Mater. Sci.* **29**, 53–87 (1999).

[16] Cambel, V. et al. Magnetic elements for switching magnetization magnetic force microscopy tips. *J. Magn. Magn. Mater.* **322**, 2715–2721 (2010).

[17] Kuramochi, H. et al. Advantages of CNT-MFM probes in observation of domain walls of soft magnetic materials. *Surf. Sci.* **601**, 5289–5293 (2007).

[18] Porthun, S., Abelmann, L., Vellekoop, S., Ledder, J. & Hug, H. Optimization of lateral resolution in magnetic force microscopy. *Appl. Phys. Mater. Sci. Process.* **66**, S1185–S1190 (1998).

[19] Saito, H., van den Bos, A., Abelmann, L. & Lodder, J. C. High-resolution MFM: Simulation of tip sharpening. *IEEE Trans. Magn.* **39**, 3447–3449 (2003).

[20] Tanaka, K., Ishikawa, K. & Yoshimura, M. Theoretical considerations on resolution of magnetic force microscopy using carbon nanotube probe. *J. Magn. Soc. Jpn.* **36**, 293–296 (2012).

[21] Wadas, A. & Hug, H. Models for the stray field from magnetic tips used in magnetic force microscopy. *J. Appl. Phys.* **72**, 203–206 (1992).

[22] Wolny, F. et al. Iron filled carbon nanotubes as novel monopole-like sensors for quantitative magnetic force microscopy. *Nanotechnology* **21**, 435501 (2010). 20876975 10.1088/0957-4484/21/43/435501

[23] Hug, H. J. et al. Quantitative magnetic force microscopy on perpendicularly magnetized samples. *J. Appl. Phys.* **83**, 5609–5620 (1998).

[24] Grütter, P., Mamin, H. & Rugar, D. Magnetic force microscopy (mfm). In *Scanning Tunneling Microscopy II: Further applications and related scanning techniques*, 151–207 (Springer, 1992).

[25] Van Schendel, P., Hug, H., Stiefel, B., Martin, S. & Güntherodt, H.-J. A method for the calibration of magnetic force microscopy tips. *J. Appl. Phys.* **88**, 435–445 (2000).

[26] Albrecht, T. R., Grütter, P., Horne, D. & Rugar, D. Frequency modulation detection using high-q cantilevers for enhanced force microscope sensitivity. *J. Appl. Phys.* **69**, 668–673 (1991).

[27] Guimarães, A. P. & Guimaraes, A. P. *Principles of Nanomagnetism* Vol. 7 (Springer, New York, 2009).

[28] Aharoni, A. *Introduction to the Theory of Ferromagnetism* Vol. 109 (Clarendon Press, 2000).

[29] Wang, X., Yuan, H. & Wang, X. A theory on skyrmion size. *Commun. Phys.* **1**, 31 (2018).

[30] Parkin, S. S., Hayashi, M. & Thomas, L. Magnetic domain-wall racetrack memory. *Science* **320**, 190–194 (2008). 18403702 10.1126/science.1145799

[31] Fert, A., Reyren, N. & Cros, V. Magnetic skyrmions: Advances in physics and potential applications. *Nat. Rev. Mater.* **2**, 1–15 (2017).

[32] Nagaosa, N. & Tokura, Y. Topological properties and dynamics of magnetic skyrmions. *Nat. Nanotechnol.* **8**, 899–911 (2013). 24302027 10.1038/nnano.2013.243

[33] Yu, H., Xiao, J. & Schultheiss, H. Magnetic texture based magnonics. *Phys. Rep.* **905**, 1–59 (2021).

[34] Saavedra, E., Tejo, F., Vidal-Silva, N. & Escrig, J. Magnonic key based on skyrmion clusters. *Sci. Rep.* **11**, 23010 (2021). 34836994 10.1038/s41598-021-02285-0 PMC8626437

[35] Zhang, X. et al. Skyrmion-skyrmion and skyrmion-edge repulsions in skyrmion-based racetrack memory. *Sci. Rep.* **5**, 7643 (2015). 25560935 10.1038/srep07643 PMC4284505

[36] Siddiqui, S. A. et al. Magnetic domain wall based synaptic and activation function generator for neuromorphic accelerators. *Nano Lett.* **20**, 1033–1040 (2019). 10.1021/acs.nanolett.9b04200 31888336

[37] Zhang, S. et al. Direct writing of room temperature and zero field skyrmion lattices by a scanning local magnetic field. *Appl. Phys. Lett.* **112, **132405 (2018).

[38] Ognev, A. V. et al. Magnetic direct-write skyrmion nanolithography. *ACS Nano* **14**, 14960–14970 (2020). 33152236 10.1021/acsnano.0c04748

[39] Zelent, M. et al. Skyrmion formation in nanodisks using magnetic force microscopy tip. *Nanomaterials* **11**, 2627 (2021). 34685062 10.3390/nano11102627 PMC8538463

[40] Yagil, A. et al. Stray field signatures of néel textured skyrmions in ir/fe/co/pt multilayer films. *Appl. Phys. Lett.* **112, **192403 (2018).

[41] Baćani, M., Marioni, M. A., Schwenk, J. & Hug, H. J. How to measure the local Dzyaloshinskii–Moriya interaction in skyrmion thin-film multilayers. *Sci. Rep.* **9**, 3114 (2019). 30816268 10.1038/s41598-019-39501-x PMC6395602

[42] Kravchuk, V. P., Sheka, D. D., Rößler, U. K., van den Brink, J. & Gaididei, Y. Spin eigenmodes of magnetic skyrmions and the problem of the effective skyrmion mass. *Phys. Rev. B* **97**, 064403 (2018).

[43] Rice, P. & Russek, S. E. Observation of the effects of tip magnetization states on magnetic force microscopy images. *J. Appl. Phys.* **85**, 5163–5165 (1999).

[44] Corte-León, H. et al. Comparison and validation of different magnetic force microscopy calibration schemes. *Small* **16**, 1906144 (2020). 10.1002/smll.201906144 32037728

[45] Freitag, N. H. et al. Simultaneous magnetic field and field gradient mapping of hexagonal MnNiGa by quantitative magnetic force microscopy. *Commun. Phys.* **6**, 11 (2023).

[46] Feng, Y., Mandru, A.-O., Yıldırım, O. & Hug, H. Quantitative magnetic force microscopy: Transfer-function method revisited. *Phys. Rev. Appl.* **18**, 024016 (2022).

[47] Schwarz, A. & Wiesendanger, R. Magnetic sensitive force microscopy. *Nano Today* **3**, 28–39 (2008).

[48] Goia, G. & James, R. Micromagnetics of very thin films. *Proc. R. Soc. A* **453**, 213–223 (1997).

[49] Riveros, A., Tejo, F., Escrig, J., Guslienko, K. & Chubykalo-Fesenko, O. Field-dependent energy barriers of magnetic néel skyrmions in ultrathin circular nanodots. *Phys. Rev. Appl.* **16**, 014068 (2021).

[50] Tejo, F., Riveros, A., Escrig, J., Guslienko, K. & Chubykalo-Fesenko, O. Distinct magnetic field dependence of néel skyrmion sizes in ultrathin nanodots. *Sci. Rep.* **8**, 6280 (2018). 29674646 10.1038/s41598-018-24582-x PMC5908873

[51] Vidal-Silva, N., Riveros, A., Tejo, F., Escrig, J. & Altbir, D. Controlling the nucleation and annihilation of skyrmions with magnetostatic interactions. *Appl. Phys. Lett.* **115, **082405 (2019).

[52] Huo, S. et al. Micromagnetic and mfm studies of a domain wall in thick 1 1 0 fesi. *J. Magn. Magn. Mater.* **190**, 17–27 (1998).

[53] Prejbeanu, I., Buda, L., Ebels, U. & Ounadjela, K. Observation of asymmetric bloch walls in epitaxial co films with strong in-plane uniaxial anisotropy. *Appl. Phys. Lett.* **77**, 3066–3068 (2000).

[54] Foss, S., Proksch, R., Dahlberg, E. D., Moskowitz, B. & Walsh, B. Localized micromagnetic perturbation of domain walls in magnetite using a magnetic force microscope. *Appl. Phys. Lett.* **69**, 3426–3428 (1996).

[55] Sader, J. E. & Jarvis, S. P. Accurate formulas for interaction force and energy in frequency modulation force spectroscopy. *Appl. Phys. Lett.* **84**, 1801–1803 (2004).

[56] Giessibl, F. J. Forces and frequency shifts in atomic-resolution dynamic-force microscopy. *Phys. Rev. B* **56**, 16010 (1997).

[57] Casiraghi, A. et al. Individual skyrmion manipulation by local magnetic field gradients. *Commun. Phys.* **2**, 145 (2019).

[58] Tan, A. K. et al. Skyrmion generation from irreversible fission of stripes in chiral multilayer films. *Phys. Rev. Mater.* **4**, 114419 (2020).

[59] Schryer, N. L. & Walker, L. R. The motion of 180 domain walls in uniform dc magnetic fields. *J. Appl. Phys.* **45**, 5406–5421 (1974).

[60] Jiang, A., Zhou, Y., Zhang, X. & Mochizuki, M. Transformation of a skyrmionium to a skyrmion through the thermal annihilation of the inner skyrmion. *Phys. Rev. Res.* **6**, 013229 (2024).

[61] Cheng, R. et al. Magnetic domain wall skyrmions. *Phys. Rev. B* **99**, 184412 (2019).

[62] Riveros, A. et al. Magnetic vortex core in cylindrical nanostructures: Looking for its stability in terms of geometric and magnetic parameters. *J. Magn. Magn. Mater.* **401**, 848–852 (2016).

[63] Yin, L. et al. Magnetocrystalline anisotropy in permalloy revisited. *Phys. Rev. Lett.* **97**, 067203 (2006). 17026198 10.1103/PhysRevLett.97.067203

[64] DeBonte, W. Properties of thick-walled cylindrical magnetic domains in uniaxial platelets. *J. Appl. Phys.* **44**, 1793–1797 (1973).

[65] Giordano, A., Finocchio, G., Torres, L., Carpentieri, M. & Azzerboni, B. Semi-implicit integration scheme for Landau–Lifshitz–Gilbert–Slonczewski equation. *J. Appl. Phys.* **111**, 15 (2012).

