# Supplementary material for: Modeling the spatial resolution of magnetic solitons in magnetic force microscopy and the effect on their sizes
Source: Sci Rep. 2025 Apr 8;15:11944. doi: 10.1038/s41598-025-95584-9 (PMC11978781; doi:10.1038/s41598-025-95584-9)
Supplement: Supplementary file 1 — Supplementary Information. [file 41598_2025_95584_MOESM1_ESM.pdf]

**SUPPLEMENTARY INFORMATION**

# Modeling the spatial resolution of magnetic solitons in Magnetic Force Microscopy and the effect on their sizes


I. Castro and N. Vidal-Silva

*Departamento de Ciencias Físicas, Universidad de La Frontera, Casilla 54-D, 4811186 Temuco, Chile.*

A. Riveros and J. L. Palma

*Escuela de Ingeniería, Universidad Central de Chile, Avda. Santa Isabel 1186, 8330601 Santiago, Chile*

L. Abelmann

*Delft University of Technology, Delft, The Netherlands*

R. Tomasello and D. R. Rodrigues

*Department of Electrical and Information Engineering, Technical University of Bari, Bari, 70125, Italy.*

A. Giordano

*Department of Engineering, University of Messina, 98166, Italy.*

G. Finocchio

*Department of Mathematical and Computer Sciences, Physical Sciences and Earth Sciences, University of Messina, Messina, I-98166, Italy.*

R. A. Gallardo

*Universidad Técnica Federico Santa María, Avenida España 1680, 2390123 Valparaiso, Chile*



(Dated: August 22, 2024)

# I. MAGNETOSTATIC FIELD OF AN UNIFORMLY MAGNETIZED SPHERICAL TIP

The picture of the coordinates system from sample and tip center is depicted in Fig. A1.

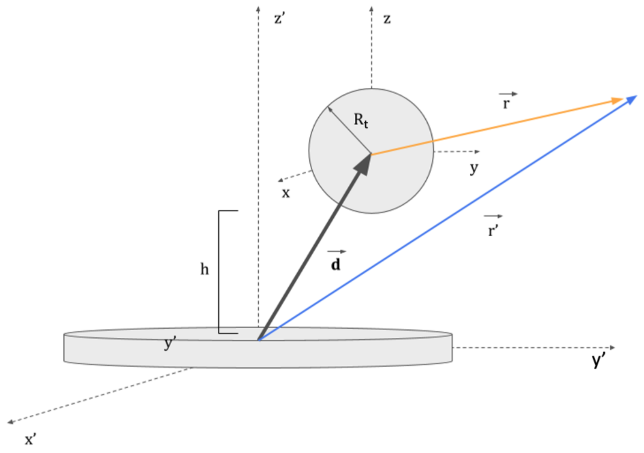


FIG. A1. Schematic representation of the system studied. The frame $x'y'z'$ is fixed at the sample (which corresponds to a nanodisk or a nanostrip), while the frame $xyz$ is fixed to the spherical tip

Since we have chosen to carry out the integral involved in Eq. 2 on the sample volume $V_s$, we must to express both $\mathbf{M}_s$ and $\mathbf{H}_t$ in a coordinate system solidarity to the sample. We start by computing the magnetostatic field generated by the spherical tip as seen in a reference frame fixed at its origin. In this case, $\mathbf{H}_t = -\nabla U(\mathbf{r})$, with the magnetostatic potential

$$U(\mathbf{r}) = \frac{1}{4\pi}\left[-\int_{V'} \frac{\nabla' \cdot \mathbf{M}(\mathbf{r}')}{|\mathbf{r}-\mathbf{r}'|}dv' + \int_{S'} \frac{\mathbf{M}(\mathbf{r}')\cdot\hat{n}'}{|\mathbf{r}-\mathbf{r}'|}ds'\right], \tag{S.1}$$

where $V'$ ($S'$) stands for the sphere volume (surface). Considering the homogeneous magnetization of tip pointing to the $\pm z$ direction, the volume integral vanishes. On the other hand, the spherical symmetry can be exploited to expand the Green function $|\mathbf{r}-\mathbf{r}'|^{-1}$ as

$$\frac{1}{|\mathbf{r}-\mathbf{r}'|} = \sum_{l=1}^{\infty}\sum_{m=-l}^{l} \frac{4\pi}{2l+1}\frac{r_<^l}{r_>^{l+1}} Y_{l,m}(\theta,\phi)Y_{l,-m}(\theta',\phi'), \tag{S.2}$$

where $Y_{l,m}(\theta,\phi)$ are the spherical harmonic functions, which are given by

$$Y_{l,m}(\theta,\phi) = \sqrt{\frac{2l+1}{4\pi}\frac{(l-m)!}{(l+m)!}}\, P_l^m(\cos\theta)e^{im\phi}, \tag{S.3}$$

with $P_l^m(\cos\theta)$ the Legendre Polynomials. Since in our case $\hat{n}' = \hat{r}'$, the magnetostatic potential can be written as follows

$$U(\mathbf{r}) = \frac{M_t}{4\pi}\sum_{l=1}^{\infty}\sum_{m=-l}^{l}\frac{r_<^l}{r_>^{l+1}}\, P_l^m(\cos\theta)e^{im\phi}\int_{S'}\cos\theta'\, P_l^{-m}(\cos\theta')e^{-im\phi'}\, ds' \tag{S.4}$$

By using the identity $2\pi\,\delta_{0,m} = \int_0^{2\pi} e^{-im\phi'}\, d\phi'$, and exploiting the orthogonality of Legendre Polynomials, we get the following expression for the magnetostatic potential

$$U(\mathbf{r}) = \frac{M_t}{3}\frac{r_<}{r_>^2}\, R_t^2\cos\theta \tag{S.5}$$

As described above, we need to find an expression for $\mathbf{H}_t$ on the sample reference system. To do that, we start by calculating the magnetostatic potential in the region outer the sphere as seen on its own reference system. Thus, in this region $R_t < r$, the magnetostatic potencial is given by $U(\mathbf{r}) = \frac{M_t R^3}{3}\,\frac{\cos\theta}{r^2}$, from which we can formally get the magnetostatic field

$$\mathbf{H}_t(\mathbf{r}) = \frac{M_t R_t^3}{3r^3}[2\cos\theta\hat{r} + \sin\theta\hat{\theta}], \tag{S.6}$$

that corresponds to Eq. (5) of the main text. This expression can be written in cartesian coordinates

$$\mathbf{H}_t(\mathbf{r}) = \frac{M_t R_t^3}{(x^2+y^2+z^2)^{5/2}}\left[xz\hat{x} + yz\hat{y} + \frac{1}{3}(2z^2 - x^2 - y^2)\,\hat{z}\right] \tag{S.7}$$

By using the following transformation between sample and tip reference frames (see Fig. A1), $x' = x$ , $y' = y + d_y$, $z' = z + d_z$, where $d_y$ and $d_z$ are the components of the $\mathbf{d}$ vector mentioned in the main text, we can find the expression for $\mathbf{H}_t$ in the sample reference system. The expression for the stray field in cartesian and cylindrical coordenates is given by

$$\begin{aligned}\mathbf{H}_t(\mathbf{r}') =& \frac{M_t R_t^3}{(x'^2 + (y'-d_y)^2 + (z'-d_z)^2)^{5/2}} \\ & \left[x'(z'-d_z)\hat{x}' + (y'-d_y)(z'-d_z)\hat{y}'' + \frac{1}{3}(2(z'-d_z)^2 - x'^2 - (y'-d_y)^2)\,\hat{z}'\right]\end{aligned} \tag{S.8}$$

$$\begin{aligned}\mathbf{H}_t(\mathbf{r}') =& \frac{M_t R_t^3}{(\rho'^2\cos^2\phi' + (\rho'\sin\phi' - d_y)^2 + (z'-d_z)^2)^{5/2}}\Big((\rho' - d_y\sin\phi')(z'-d_z)\,\hat{\rho}' \\ & - d_y\cos\phi'(z'-d_z)\,\hat{\phi}' + \frac{1}{3}(2(z'-d_z)^2 - \rho'^2\cos^2\phi' - (\rho'\sin\phi' - d_y)^2)\,\hat{z}'\Big)\end{aligned} \tag{S.9}$$

It can be easily noted that if one want to considerate the opposite direction $+z$ for the tip magnetization, these results will only change in a global sign.

## II. TOTAL MICROMAGNETIC ENERGY OF THE SAMPLE AND MAGNETIC FORCE

The total micromagnetic energy comprises the exchange, magnetostatic, effective anisotropy (including dipolar energy), Dzyaloshiinski-Moriya, and interaction energy. The latter is obtained by integrating the dot point of Eq. (A7) or (A8) with the sample magnetization. The rest of energies can be calculated as

$$E_{\text{ex}} = A_{\text{ex}} \int \sum_i (\nabla m_i)^2 \, dV_s, \tag{S.10}$$

where $A_{\text{ex}}$ is the stiffness constant, and $m_i$ is the ith-component of the sample magnetization with $i = x, y, z$. The magnetic anisotropy energy comprises the contribution from an easy axis (proportional to $K_u$) and an easy plane, which emerges from a magnetostatic approximation proportional to $M_s^2$

$$E_{\text{ani}} = -K_{\text{eff}} \int m_z^2 \, dV_s, \tag{S.11}$$

where $K_{\text{eff}} = K_u - \mu_0 M_s^2/2$ is the effective anisotropy constant, with $K_u$ the uniaxial anisotropy constant, and the second term corresponds to an approximation to the dipolar interaction of the sample. Finally, the interfacial DMI energy is given by

$$E_{\text{DM}} = -D \int [m_z \vec{\nabla} \cdot \vec{m} - (\vec{m} \cdot \vec{\nabla})\, m_z] \, dV_s, \tag{S.12}$$

where $D$ is the DMI parameter.

### A. Domain Walls

For the case of DWs, it is convenient to use the expression for $\mathbf{H}_t$ written in cartesian coordinates. By considering the following ansatz for the DW magnetization field

$$m_y(y, \Delta) = \tanh\left(\frac{y - y_0}{\Delta}\right), \tag{S.13}$$

we arrive to the interaction energy

$$E_{\text{int}} = \mp \frac{\mu_0 M_s M_t L_x L_z R_t^3}{3} \int_{-\infty}^{\infty} \frac{m_z(y,\Delta)(2d_z^2 - (y-d_y)^2) - 3d_z(y-d_y)m_y(y,\Delta)}{((y-d_y)^2 + d_z^2)^{5/2}} dy, \tag{S.14}$$

where the negative sign will be considered if $\mathbf{M}_t$ is pointing in the $+z$ direction, and the positive sign will be considered if $\mathbf{M}_t$ is pointing in the opposite $-z$ direction. Next, we can now calculate the $z$ component of the magnetic force on the sample and its derivative respect to the $d_z$ distance, for $d_y = 0$ it holds:

$$F_z(\Delta) = \pm \mu_0 M_s M_t R_t^3 L_x L_z \int_{-\infty}^{\infty} dy \frac{m_y(y,\Delta)\, y(y^2 - 4d_z^2) + d_z(2d_z^2 - 3y^2)\, m_z(y,\Delta)}{(y^2 + d_z^2)^{7/2}}, \tag{S.15}$$

while for the general case $d_y \neq 0$:

$$F_z(\Delta) = \pm \mu_0 M_s M_t R_t^3 L_x L_z \int_{-\infty}^{\infty} \left[ \frac{m_y(y,\Delta)\,(y-d_y)((y-d_y)^2 - 4d_z^2)}{((y-d_y)^2 + d_z^2)^{7/2}} + \frac{d_z(2d_z^2 - 3(y-d_y)^2)\, m_z(y,\Delta)}{((y-d_y)^2 + d_z^2)^{7/2}} \right] dy, \tag{S.16}$$

and the derivative of the force over the sample for the general case can be written as

$$\frac{\partial F_z}{\partial d_z} = \pm \mu_0 M_s M_t R_t^3 L_x L_z \int_{-\infty}^{\infty} \left[ \frac{m_y(y,\Delta)(20d_z^3(y-d_y) - 15d_z(y-d_y)^3)}{((y-d_y)^2 + d_z^2)^{9/2}} + \frac{m_z(y,\Delta)(24d_z^2(y-d_y)^2 - 8d_z^4 - 3(y-d_y)^4)}{((y-d_y)^2 + d_z^2)^{9/2}} \right] dy \tag{S.17}$$

By using the ansatz (13), the exchange and magnetic anisotropy energies reads

$$E_{ex} = \frac{2A_{\text{ex}} L_x L_z}{\Delta}, \tag{S.18}$$

$$E_{\text{ani}} = -2K_{\text{eff}} L_x L_z \Delta. \tag{S.19}$$

### B. Skyrmions

For this magnetic texture we must proceed analogously to the previous case. In this case we use the following ansatz

$$m_z(\rho, r_s) = -P\cos[2\arctan(f(\rho, r_s))] = -P\cos[2\arctan((r_s/\rho)\exp(\xi(r_s - \rho)/l_{\text{ex}})], \tag{S.20}$$

whose parameters are introduced in the main text. Due to the symmetry of the nanodisk, it is convenient to use the expression for $\mathbf{H}_t$ in cylindrical coordinates. The interaction energy between the tip and sample can be written as follows

$$E_{\text{int}} = \mp \frac{\mu_0 M_s M_t R_t^3}{3} \int_0^{L_z} \int_0^{2\pi} \int_0^{R_s} \frac{\rho}{(\rho^2 \cos^2\phi + (\rho\sin\phi - d_y)^2 + (z-d_z)^2)^{5/2}} \times \Big[ 3 m_\rho(\rho, r_s)(\rho - d_y \sin\phi)(z - d_z) + m_z(\rho, r_s)\left(2(z-d_z)^2 - \rho^2\cos^2\phi - (\rho\sin\phi - d_y)^2\right) \Big] d\rho\, d\phi\, dz. \tag{S.21}$$

Therefore, the $z$ component of the magnetic force on the sample for $d_y = 0$, which corresponds to Eq.(4) of the main article, is given by

$$F_z(r_s) = \pm 2\pi\mu_0 M_s M_t R_t^3 \left[ \int_0^{L_z} \int_0^{R_s} \frac{\rho^2\, d\rho\, dz\, (\rho^2 - 4(z-d_z)^2)\, m_\rho(\rho, r_s)}{(\rho^2 + (z-d_z)^2)^{7/2}} + \int_0^{L_z} \int_0^{R_s} \frac{\rho\, d\rho\, dz\, (z-d_z)\,(3\rho^2 - 2(z-d_z)^2)\, m_z(\rho, r_s)}{(\rho^2 + (z-d_z)^2)^{7/2}} \right], \tag{S.22}$$

while the general case for $d_y \neq 0$ reads

$$F_z = \pm \mu_0 M_s M_t R_t^3 \int_0^{2\pi} \int_0^{L_z} \int_0^{R_s} \frac{\rho\, d\rho\, dz d\phi}{(\rho^2\cos^2\phi + (\rho\sin\phi - d_y)^2 + (z-d_z)^2)^{7/2}} \times \Big[ (\rho - d_y\sin\phi) \left[\rho^2\cos^2\phi + (\rho\sin\phi - d_y)^2 - 4(z-d_z)^2\right] m_\rho(\rho, r_s) + (z-d_z) \left[3\rho^2\cos^2\phi + 3(\rho\sin\phi - d_y)^2 - 2(z-d_z)^2\right] m_z(\rho, r_s) \Big]. \tag{S.23}$$

The derivative for the general case reads

$$\frac{\partial F_z}{\partial d_z} = \pm \mu_0 M_s M_t R_t^3 \int_0^{2\pi} \int_0^{L_z} \int_0^{R_s} \frac{d\rho\, dz d\phi}{(\rho^2\cos^2\phi + (\rho\sin\phi - d_y)^2 + (z-d_z)^2)^{9/2}} \times \Big[ 5(\rho - d_y\sin\phi)(z-d_z) \left[3\rho^2\cos^2\phi + 3(\rho\sin\phi - d_y)^2 - 4(z-d_z)^2\right] m_\rho(\rho, r_s) + \left[(24\rho^2\cos^2\phi(z-d_z)^2 + 24(\rho\sin\phi - d_y)^2(z-d_z)^2 - 8(z-d_z)^4 - 3\rho^4\cos^4\phi - 3(\rho\sin\phi - d_y)^4 - 6\rho^2\cos^2\phi(\rho\sin\phi - d_y)^2\right] m_z(\rho, r_s) \Big] \tag{S.24}$$

Finally, the expressions for exchange energy, anisotropy, and DMI are explicitly given replacing the skyrmion ansatz in Eq. (S10-12). In cylindrical coordinates, the exchange energy reads

$$
\begin{aligned}
E_{\mathrm{ex}} = 2\pi L_z A_{\mathrm{ex}} \int_0^{R_s} \Bigg[ \Bigg( \frac{1}{\rho}(1 - P^2 \cos^2[2\arctan((r_s/\rho)\exp(\xi(r_s-\rho)/l_{\mathrm{ex}})]) \Bigg) \\
+ \frac{4P^2 r_s^2 \exp^2(\frac{\xi}{l_{\mathrm{ex}}}(r_s-\rho))(\frac{1}{\rho}+\frac{\xi}{l_{\mathrm{ex}}})^2 \sin^2(2\arctan(\frac{r_s}{\rho}\exp(\frac{\xi}{l_{\mathrm{ex}}}(r_s-\rho))))}{\rho\,(1+(\frac{r_s}{\rho}\exp(\frac{\xi}{l_{\mathrm{ex}}}(r_s-\rho)))^2)^2\,(1-P^2\cos^2[2\arctan((r_s/\rho)\exp(\xi(r_s-\rho)/l_{\mathrm{ex}})])} \Bigg] d\rho,
\end{aligned}
\tag{S.25}
$$

Next, the effective anisotropy energy can be easily calculated

$$
E_{\mathrm{ani}} = -2\pi L_z K_{\mathrm{eff}} \int_0^{R_s} \rho\, P^2 \cos^2[2\arctan((r_s/\rho)\exp(\xi(r_s-\rho)/l_{\mathrm{ex}})]\, d\rho \tag{S.26}
$$

Finally, the DMI is

$$
\begin{aligned}
E_{\mathrm{DM}} = -2\pi L_z D \int_0^{R_s} \Bigg[ \frac{2P^2\cos^2[2\arctan((r_s/\rho)\exp(\xi(r_s-\rho)/l_{\mathrm{ex}})]-1}{\sqrt{1-P^2\cos^2[2\arctan((r_s/\rho)\exp(\xi(r_s-\rho)/l_{\mathrm{ex}})]}} \\
\frac{2Pr_s\exp(\frac{\xi}{l_{\mathrm{ex}}}(r_s-\rho))(\frac{1}{\rho}+\frac{\xi}{l_{\mathrm{ex}}})\,\sin(2\arctan(\frac{r_s}{\rho}\exp(\frac{\xi}{l_{\mathrm{ex}}}(r_s-\rho))))}{1+(\frac{r_s}{\rho}\exp(\frac{\xi}{l_{\mathrm{ex}}}(r_s-\rho)))^2} \\
+ P\cos[2\arctan((r_s/\rho)\exp(\xi(r_s-\rho)/l_{\mathrm{ex}})] \\
\times \sqrt{1-P^2\cos^2[2\arctan((r_s/\rho)\exp(\xi(r_s-\rho)/l_{\mathrm{ex}})]} \Bigg] d\rho.
\end{aligned}
\tag{S.27}
$$

## III. CALCULATION OF $l_{\mathrm{th}}$

Here we show the numerical solution of Eq. (1) of the main text by matching both sides of it. For the DW case, we depict the numerical evaluation of Eq. (1) as a function of $\Delta$ by matching the left-hand side (solid curves) with the right-hand one (dashed curves) for selected distances of separation $h$ and tip radii $R_t$. According to Eq. (1), the corresponding $l_{\mathrm{th}}$ can be extracted from the value of $\Delta$ where both solid and dashed curves intersect. It is important to point out that, due to the shape of solid curves, there are more than one $\Delta$ at which the curves match. However, as previously done in Refs. [1–7] , we only consider those points that satisfy the fact that the $l_{\mathrm{th}}$ must increase with the temperature.

The skyrmion case is shown in Fig. A3. Fig. A3a) corresponds to the solution for different heights of separation $h$ and a fixed $R_t = 5$ nm, while in Fig. A3a) the height is fixed to $h = 21$ nm, and the solution is plotted for different tip radii $R_t$. Similar to the DW case, we consider those values of $2r_s = l_{\mathrm{th}}$ that satisfy the fact of becoming larger as the temperature increases.

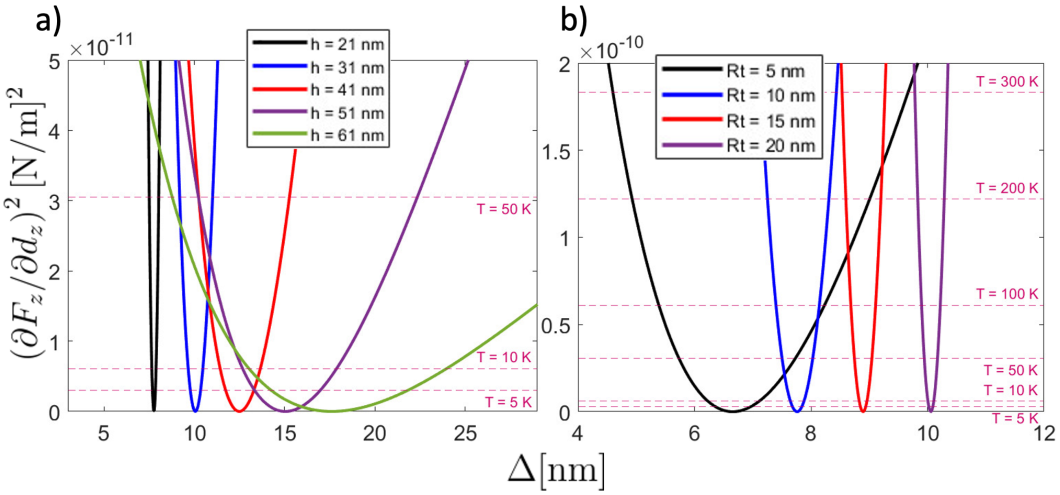


FIG. A2. Numerical evaluation of Eq. (1). The solid curves correspond to the left-hand side of Eq. (1) as a function of the DW width $\Delta$ for a) $R_t = 10$ nm and different lengths of separation $h$; and b) $h = 21$ nm for different tip radius $R_t$. The pink dashed line corresponds to the right-hand side of Eq. (1) for different temperatures. The value of $\Delta$ at which the solid and dashed lines intersect corresponds to $l_{\text{th}}$.

## IV. VARIATION OF THE DW WIDTH WITH THE EXTERNAL MAGNETIC FIELD AND LATERAL DISTANCE $d_y$

Here we show the variation of the DW width that minimized the total magnetic energy as a function of tip lateral distance and as function of an uniform external magnetic field applied in the $z-$direction. As can be seen from Fig. A4, the DW width enhances when it points in the same direction as the magnetic field. This enhancement allows us to simulate the remanence state in real experiments.

The effect of tip on the DW width is shown in Fig. A5, where we show $l_{\text{ph}}$, as a function of the height of separation under an applied field of 340 mT for repulsive configuration A5a,c), and attractive configuration Figs. A5b,d). In this case there exist a larger DW deformations according to the relative configuration between the sample and the tip's magnetization. For example, when the tip points parallel to the DW magnetization (attractive configuration), and a tip radius of 40 nm is employed in measurement at $h = 40$ nm, the resulting DW width is about 80 nm, representing an increment of about 60%. While, when the tip points opposite to the DW magnetization (repulsive configuration) at same tip radius and height, the resulting DW width is about 15 nm, representing a decrement of about 70%. This behavior is also observed in real measurements. Indeed, Ref.

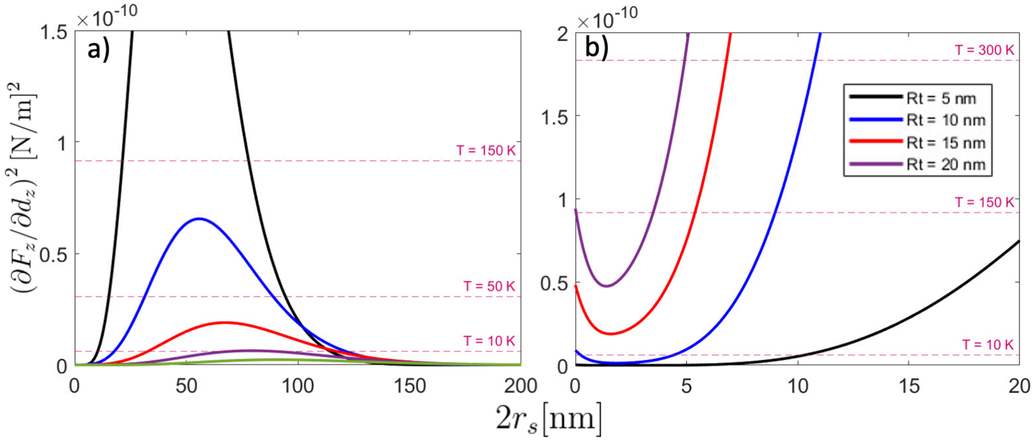


FIG. A3. Numerical evaluation of Eq. (1) for the skyrmion case. The solid curves correspond to the left-hand side of Eq. (1) as a function of the skyrmion diameter $2r_s$ for a) $R_t = 5$ nm and different lengths of separation $h$; and b) $h = 21$ nm for different tip radius $R_t$. The pink dashed line corresponds to the right-hand side of Eq. (1) at distinct temperatures. The value of $2r_s$ at which the solid and dashed lines intersect corresponds to $l_{\text{th}}$.

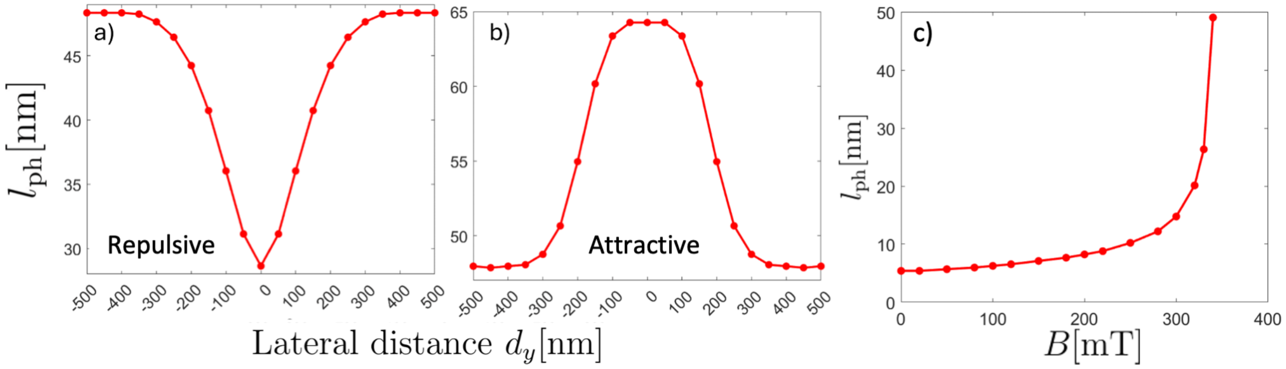


FIG. A4. DW in stripes: Physical length $l_{\text{ph}}$ as a function of the horizontal component of the distance of separation $\mathbf{d}$ for a) $\mathbf{m}_t = -\hat{k}$ and b) $\mathbf{m}_t = +\hat{k}$, at fixed $h = 15$ nm and for $R_{\text{t}} = 20$ nm. c) Physical length $l_{\text{ph}}$ as a function of an external magnetic field.

[8] show an increment of approximately 60% of DW width in attractive mode respect to repulsive repulsive one in samples with similar sizes as presented here. This variation qualitatively agrees with the results shown above, and as we claim here, the deformation degree can vary according to the tip geometry and the height of separation at which the measurement is carried out. Finally, we calculate $l_{\text{ph}}$ as a function of the lateral distance $d_y$ keeping a fixed $h$. We assumed that the

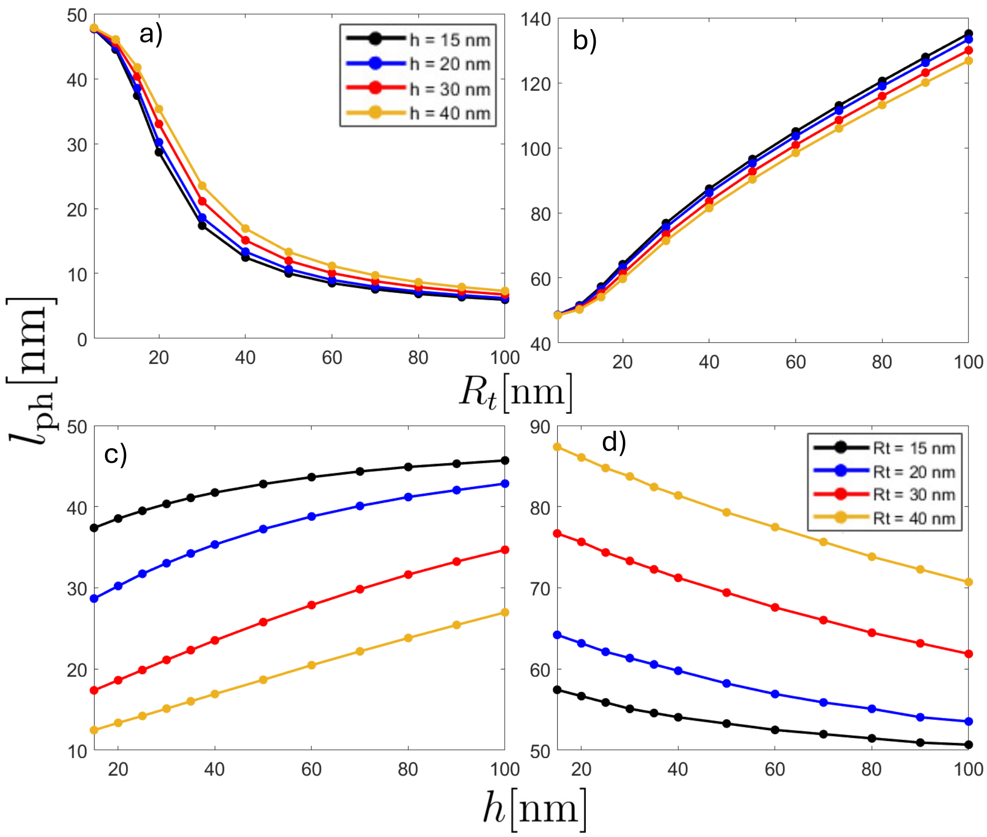


FIG. A5. (Upper panel) Physical length $l_{\mathrm{ph}}$ of the DW as a function of the tip radius for different height of separation for a) $\mathbf{m}_t = -\hat{k}$ and b) $\mathbf{m}_t = +\hat{k}$. (Lower panel) Physical length $l_{\mathrm{ph}}$ of the DW as a function of the distance of separation for different tip radii and considering tip magnetization direction c) $\mathbf{m}_t = -\hat{k}$ and d) $\mathbf{m}_t = +\hat{k}$. All cases were calculated under the application of a magnetic field of $\mathbf{B} = 340$ mT$\hat{k}$.

DW is pinned at $d_y = 0$, so there are no changes in the position of the wall. Figs. A4a,b) show the physical DW width, $l_{\mathrm{ph}}$, as a function of the lateral position of the tip with respect to the DW position at a fixed height of $h = 15$ nm using a tip of radius 20 nm in the repulsive and attractive mode, respectively.

## V. MFM SIGNAL OF A SPHERICALLY MODELED TIP COMPARED WITH THE CORRESPONDING SIGNAL OF A MODELED TIP WITH VARIABLE RADIUS

As the major contribution to the signal is from the tip apex [9], the magnetic tip could be modeled spherically. To show it we have compared in Fig. A6 a) the MFM signal $\partial F_z/\partial z$ on the tip in the presence of the skyrmion texture by using our model considering a spherical tip for

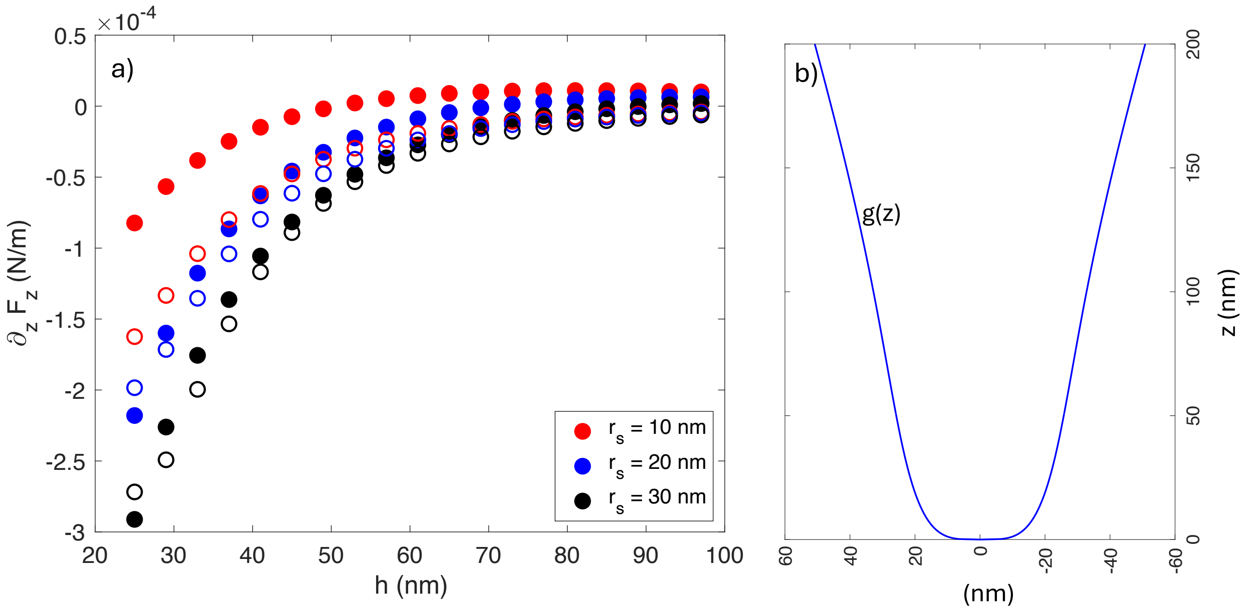


FIG. A6. a) Vertical gradient of the force over the tip as a function of the tip vertical position due to a skyrmion texture for different skyrmion radii $r_s$. Filled markers were obtained using a spherical tip model of radius 20 nm, while open markers were obtained using the complex tip model with variable radius $g(z)$ along the tip symmetry axis (using Eq.(6) of Ref. [9]). b) Profile of the tip variable radius $g(z)$ used in Ref. [9] for modeling the tip.

different skyrmion radii (filled markers) with the corresponding MFM signal of a tip modeled with variable radius $g(z)$ along the tip symmetry axis used in reference [9] (open markers). It can be seen that the spherical tip model is in the same order and shows similar behavior to that of the more complex tip geometry (open markers). We have used here a spherical tip radius of 20 nm since it is approximately the curvature radius given by the $g(z)$ function at the proximity to the tip apex (see Figure A6 b)). Therefore, the spherical tip should capture most of the physical behavior despite its simplicity.

---


[1] L. Abelmann, A. van den Bos, and C. Lodder, Magnetic Microscopy of Nanostructures, 2005th Edition, Springer , 254 (2005).

[2] K. Tanaka, K. Ishikawa, and M. Yoshimura, Journal of the Magnetics Society of Japan **36**, 293 (2012).

[3] S. Porthun, L. Abelmann, and C. Lodder, Journal of magnetism and magnetic materials **182**, 238 (1998).

[4] S. Porthun, L. Abelmann, S. Vellekoop, J. Ledder, and H. Hug, APPLIED PHYSICS A MATERIALS SCIENCE AND PROCESSING **66**, S1185 (1998).

[5] H. Saito, A. van den Bos, L. Abelmann, and J. C. Lodder, IEEE transactions on magnetics **39**, 3447 (2003).

[6] C. Schönenberger and S. Alvarado, Zeitschrift für Physik B Condensed Matter **80**, 373 (1990).

[7] H. J. Hug, B. Stiefel, P. Van Schendel, A. Moser, R. Hofer, S. Martin, H.-J. Güntherodt, S. Porthun, L. Abelmann, J. Lodder, et al., Journal of Applied Physics **83**, 5609 (1998).

[8] I. Prejbeanu, L. Buda, U. Ebels, and K. Ounadjela, Applied Physics Letters **77**, 3066 (2000).

[9] A. Yagil, A. Almoalem, A. Soumyanarayanan, A. K. Tan, M. Raju, C. Panagopoulos, and O. Auslaender, Applied Physics Letters **112** (2018).
